# Nutritional Status, Dietary Intake and Immunosuppressive Therapy in Kidney Transplant Recipients: Differences Between Early and Late Post-Transplant Period—A Cross-Sectional Pilot Study

**DOI:** 10.3390/nu18162725

**Published:** 2026-08-20

**Authors:** Aleksandra Anna Kajdas, Dorota Szostak-Węgierek, Krzysztof Krasuski, Dorota Miszewska-Szyszkowska, Magdalena Durlik

**Affiliations:** 1 Department of Clinical Dietetics, Medical University of Warsaw, Erazma Ciolka 27, 01-445 Warsaw, Poland; dorota.szostak-wegierek@wum.edu.pl; 2 Department of Medical Informatics and Telemedicine, Medical University of Warsaw, Litewska 14/16, 00-581 Warsaw, Poland; krzysztof.krasuski@wum.edu.pl; 3 Faculty of Mathematics and Informatics Science, Warsaw University of Technology, Koszykowa 75, 00-662 Warsaw, Poland; 4 Department of Transplantology, Immunology, Nephrology and Internal Diseases, Medical University of Warsaw, Nowogrodzka 59, 02-014 Warsaw, Poland; dorota.szyszkowska@uckwum.pl (D.M.-S.); magdalena.durlik@wum.edu.pl (M.D.)

**Keywords:** kidney transplant recipients, dietary intake, nutritional assessment, body composition, bioelectrical impedance analysis, immunosuppressive therapy, tacrolimus

## Abstract

Background/Objectives: Kidney transplantation is associated with profound metabolic and nutritional changes, particularly across different post-transplant periods. This study aimed to evaluate differences in nutritional status, dietary intake, and immunosuppressive therapy between recipients in the early and late post-transplant period. Materials and Methods: This cross-sectional pilot study presents an analysis of baseline data obtained from a prospective cohort of 38 kidney transplant recipients divided into early (≤1 year post-transplantation; *n* = 15) and late (>1 year post-transplantation; *n* = 23) post-transplant groups. Nutritional status was assessed using anthropometric and laboratory measurements, bioelectrical impedance analysis (BIA), and Nutritional Risk Screening 2002 (NRS-2002). Dietary intake was assessed using the Food Frequency Questionnaire-6 (FFQ-6) and a 3-day dietary record. Information regarding immunosuppressive therapy was obtained from medical records and verified at routine clinic visits. Results: The groups did not differ significantly with respect to sociodemographic characteristics, comorbidities, anthropometric measurements, body composition, and nutritional risk. No significant differences were observed in body weight, body mass index (BMI), body composition parameters, or most laboratory measurements. Early post-transplant recipients exhibited significantly higher non-high-density lipoprotein (non-HDL) cholesterol concentrations (153.3 ± 37.5 vs. 111.1 ± 50.8 mg/dL; *p* = 0.040), higher tacrolimus trough concentrations (11.2 ± 5.2 vs. 4.6 ± 1.1 ng/mL; *p* = 0.0001), and lower serum magnesium concentrations (1.64 ± 0.13 vs. 1.94 ± 0.15 mg/dL; *p* = 0.009) than late post-transplant recipients. No statistically significant differences were observed for most assessed dietary habits, except for the more frequent addition of sugar to beverages among the early post-transplant group (*p* = 0.048). Qualitative assessment of 3-day dietary records showed a tendency toward more regular meals and greater dietary variety among early post-transplant recipients. This group also received significantly higher tacrolimus trough concentrations and higher prednisone-equivalent and mycophenolate doses than recipients in the late post-transplant period. Conclusions: In this pilot study, the observed differences between early and late post-transplant recipients concerned primarily immunosuppressive exposure and selected metabolic parameters, while no statistically significant differences were identified for most measures of nutritional status or dietary intake. Given the small sample size and the exploratory nature of the study, these findings should be considered preliminary and hypothesis-generating rather than confirmatory. Larger, adequately powered prospective studies are needed to validate these observations and further evaluate nutritional and metabolic changes across the post-transplant course.

## 1. Introduction

Kidney transplantation improves survival and quality of life in patients with end-stage renal disease and partially reverses the metabolic abnormalities associated with chronic kidney disease and dialysis [1,2]. However, kidney transplant recipients remain at risk of nutritional and metabolic complications including weight gain, excess adiposity, and metabolic disturbances, which may affect long-term health outcomes [3].

Nutritional status after kidney transplantation is influenced by several interacting factors, including recovery from dialysis-related catabolism, dietary habits, graft function, physical activity, and immunosuppressive therapy [4]. The relative contribution of these factors may differ according to post-transplant period. The early post-transplant period is characterized by more intensive immunosuppressive treatment, particularly corticosteroid-based therapy, which may contribute to metabolic disturbances and changes in body composition [5,6]. In contrast, recipients in the later post-transplant period generally receive lower maintenance doses, although long-term exposure to immunosuppressive agents may still be associated with metabolic complications [7,8]. Dietary intake may also change after transplantation as dietary restrictions are generally reduced. However, adherence to healthy dietary recommendations remains suboptimal in many recipients [9,10].

Although previous studies have examined individual aspects of nutrition and metabolism after kidney transplantation, evidence integrating nutritional status, dietary intake, body composition, biochemical parameters, and immunosuppressive therapy across different post-transplant periods remains limited [5,10,11,12,13]. Comparing these factors between early and late post-transplant recipients may provide a broader description of nutritional and metabolic characteristics at different stages after transplantation.

Therefore, this pilot study aimed to evaluate differences in dietary intake, nutritional status including body composition, and immunosuppressive therapy between kidney transplant recipients in the early (≤1 year) and late (>1 year) post-transplant periods.

## 2. Materials and Methods

### 2.1. Study Design and Participants

This study presents a cross-sectional analysis of baseline data obtained from a prospective cohort of 38 kidney transplant recipients divided into early (≤1 year post-transplantation) (*n* = 15) and late (>1 year post-transplantation) (*n* = 23) post-transplant groups. Participants were recruited at the Transplant Medicine and Nephrology Clinic in Warsaw, Poland, between November 2023 and July 2024. The study was designed and reported in accordance with the Strengthening the Reporting of Observational Studies in Epidemiology (STROBE) Statement for cross-sectional studies [14], available as Supplementary Materials File S1. The one-year cutoff was defined a priori to distinguish recipients within the first year after transplantation from those in the subsequent long-term follow-up period [8,15,16]. In addition, as this was an exploratory pilot study based on consecutive eligible kidney transplant recipients recruited during the study period, a formal a priori sample size calculation was not performed.

The inclusion criteria were age ≥ 18 years, stable graft function, and written informed consent to participate in the study. Patients were required to be under regular outpatient nephrological follow-up and able to complete nutritional questionnaires and dietary interviews independently.

The exclusion criteria included acute infection, hospitalization during the month preceding enrollment, active malignancy, pregnancy, severe cognitive impairment, inability to complete dietary assessment questionnaires, current enteral or parenteral nutrition, and acute graft rejection requiring intensified immunosuppressive treatment.

All participants completed a standardized nutritional and medical interview covering demographic characteristics, medical history, transplantation-related variables, dietary habits, lifestyle factors, comorbidities, pharmacotherapy, and recent body weight changes [Supplementary Materials File S2]. The interview was conducted by a dietitian during an outpatient consultation and included information on age, sex, time since transplantation, type of transplantation, dialysis vintage, comorbidities, current pharmacotherapy, immunosuppressive treatment, and recent body weight changes. Anthropometric measurements and biochemical parameters were obtained during the consultation and verified using medical documentation. Following the assessment, all participants received individualized dietary counseling together with personalized written nutritional recommendations adapted to their clinical condition, nutritional status, laboratory findings, and current stage after kidney transplantation.

Although the study protocol included longitudinal follow-up assessments conducted at baseline, 3, 6, and 9 months after transplantation, only baseline data collected during the first study visit were analyzed in the present study.

### 2.2. Nutritional Status

Nutritional status was assessed using anthropometric measurements, laboratory parameters, body composition analysis by bioelectrical impedance analysis (BIA), and Nutritional Risk Screening 2002 (NRS-2002).

#### 2.2.1. Anthropometric Measurements

Anthropometric measurements were performed during the first study visit according to standardized procedures. To minimize inter-observer variability, all anthropometric measurements were performed by the same trained investigator using a standardized measurement protocol. Circumference measurements were obtained with a non-stretchable measuring tape applied with consistent tension throughout the assessment. The assessment included body weight (kg), height (cm), body mass index (BMI; kg/m^2^), waist circumference (cm), hip circumference (cm), waist-to-hip ratio (WHR), right and left calf circumferences (cm), and mid-upper arm circumference (cm). These measurements were used to evaluate general nutritional status, body size, fat distribution, and selected indicators of peripheral muscle mass. Body weight and height were measured using standardized procedures, with participants wearing light clothing and no shoes. BMI was calculated as body weight divided by height squared (kg/m^2^). Waist circumference was measured midway between the lower rib margin and the iliac crest, whereas hip circumference was measured at the widest point over the buttocks. WHR was subsequently calculated as the ratio of waist circumference to hip circumference. Calf circumference was measured separately for the right and left lower limb at the point of greatest circumference. Mid-upper arm circumference was measured at the midpoint between the acromion and olecranon processes of the non-dominant arm.

#### 2.2.2. Laboratory Measurements

Laboratory measurements were obtained from patients’ medical records and included the most recent biochemical results available at the time of the baseline assessment. Because laboratory testing was performed as part of routine clinical follow-up, not all parameters were available for all participants. The analyzed laboratory parameters included serum creatinine concentration, *C*-reactive protein (CRP), total cholesterol, low-density lipoprotein cholesterol (LDL-C), high-density lipoprotein cholesterol (HDL-C), non-high-density lipoprotein (non-HDL) cholesterol concentrations, triglycerides, uric acid, sodium, potassium, calcium, magnesium, serum 25-hydroxyvitamin D [25(OH)D] concentration, and tacrolimus trough concentration. Inflammatory status was assessed using serum CRP concentration, whereas kidney graft function was evaluated based on serum creatinine concentration. Lipid metabolism was assessed using total cholesterol, LDL-C, HDL-C, non-HDL cholesterol, and triglyceride concentrations. Serum concentrations of sodium, potassium, calcium, and magnesium were analyzed to evaluate electrolyte status, while serum 25-hydroxyvitamin D [25(OH)D] concentrations and uric acid concentrations were also recorded. Tacrolimus trough concentration was recorded as a marker of immunosuppressive drug exposure.

#### 2.2.3. Body Composition

A comprehensive assessment of nutritional status included body composition analysis using bioelectrical impedance analysis (BIA). Measurements were performed using a Maltron BioScan 950 device (Maltron International Ltd., Rayleigh, UK) operating at four frequencies: 5, 50, 100, and 200 kHz. The examination was conducted under standardized conditions during an outpatient dietary consultation by a trained and experienced dietitian. All BIA measurements were performed by the same investigator using a standardized measurement protocol in accordance with the manufacturer’s recommendations. Participants were examined in the supine position, barefoot, and wearing light clothing, after approximately 5 min of rest. They were instructed to avoid intense physical activity, alcohol consumption, and large meals prior to the examination. Four disposable electrodes were placed on the right hand and right foot in standardized tetrapolar positions according to the manufacturer’s recommendations: one pair on the dorsal surface of the hand (wrist and metacarpal region) and one pair on the dorsal surface of the foot (ankle and metatarsal region). The analyzed body composition parameters included body fat mass (FAT, kg), body fat percentage (FAT, %), skeletal muscle mass (Muscle, kg), fat-free mass (FFM, kg), fat-free mass percentage (FFM, %), total body water percentage (TBW, %), intracellular water percentage (ICW, %), extracellular water percentage (ECW, %), and the extracellular-to-intracellular water ratio (ECW/ICW). Additional parameters included body cell mass (BCM, kg), extracellular mass (ECM, kg), protein mass (Prot, kg), and mineral content (Mnrl, kg).

#### 2.2.4. Nutritional Risk Screening 2002 (NRS-2002)

Nutritional risk was assessed using the Nutritional Risk Screening 2002 (NRS-2002), a validated screening tool recommended by the European Society for Clinical Nutrition and Metabolism (ESPEN) for the identification of patients at risk of malnutrition [17]. The NRS-2002 is widely used in both clinical practice and research as a rapid and reliable method for detecting nutritional risk and determining the need for nutritional intervention [17,18]. The assessment was performed during the baseline dietary consultation by a trained dietitian and was used as an additional indicator of nutritional status in kidney transplant recipients. The complete NRS-2002 questionnaire is provided in Supplementary Materials File S3. The screening includes an assessment of nutritional status and disease severity, taking into account recent weight loss, changes in dietary intake, BMI, and the impact of the underlying disease on nutritional requirements. In addition, one extra point is assigned to individuals aged 70 years or older to account for the increased risk of malnutrition in older adults. The total NRS-2002 score ranges from 0 to 7 points. According to the original recommendations, a score of ≥3 points indicates nutritional risk and the need for nutritional intervention, whereas a score of <3 points indicates the absence of nutritional risk [17,18]. For the purposes of the present study, participants were classified into two categories: no nutritional risk (NRS < 3) and nutritional risk present (NRS ≥ 3).

### 2.3. Dietary Assessment

Dietary intake was assessed using the Food Frequency Questionnaire (FFQ-6) and a 3-day dietary record. Nutritional interviews and dietary assessments were conducted by a trained dietitian during outpatient dietary consultation.

#### 2.3.1. FFQ-6 Questionnaire

Dietary habits were assessed using the FFQ-6, a validated Polish-language dietary assessment tool commonly used in nutritional epidemiology studies [19,20]. The FFQ-6 evaluates the habitual frequency of consumption of selected food products and food groups over the preceding 12 months. The original FFQ-6 questionnaire includes 62 food product groups categorized into several dietary sections. For the purposes of the present study, the analysis focused on selected food products relevant to the nutritional assessment of kidney transplant recipients. These included sugar added to beverages, milk and natural dairy beverages, sweetened dairy beverages, cheese, eggs, fruit, apples and pears, bananas, avocado, vegetables, carrots and peppers, potatoes, legumes, wholegrain bread, white bread, butter, sausages, red meat, poultry and rabbit meat, lean fish, fatty fish, fruit juices, and vegetable juices. Participants reported their habitual consumption frequency using a six-point scale ranging from 1 (“never or almost never”) to 6 (“several times daily”). Higher scores indicated more frequent consumption of a given food product. The obtained scores were used to compare the frequency of consumption of individual food products between recipients in the early and late post-transplant periods. The complete version of the FFQ-6 questionnaire is provided in Supplementary Materials File S4.

#### 2.3.2. 3-Day Dietary Records

In addition to the FFQ-6 questionnaire, a qualitative assessment of 3-day food records was performed. Participants were asked to complete a food record covering three days, including two weekdays and one weekend day, and to record all meals, beverages, and snacks consumed during the recording period. Prior to completing the food record, each participant received detailed instructions from a registered dietitian regarding the method of recording dietary intake. Participants were asked to document the time of consumption, meal type, food and beverage items consumed, portion sizes expressed either in grams or household measures, and any additional comments regarding food intake. They were also encouraged to provide detailed descriptions of meals, including the type of bread consumed, cooking method (e.g., boiling, baking, frying), type of fat used, and ingredients used for meal preparation. The template of the 3-day food record is provided in Supplementary Materials File S5. The completed food records were reviewed and evaluated by a dietitian during the study visit. A qualitative dietary score was developed for the present study to assess selected dietary behaviors based on predefined dietary recommendations relevant to kidney transplant recipients. The criteria and score categories were defined a priori based on current nutritional recommendations and clinical practice. This score was used as an exploratory assessment of selected dietary behaviors and does not represent a validated dietary quality index. The qualitative assessment was based on five predefined criteria reflecting selected dietary recommendations for kidney transplant recipients: (1) Consumption of 4–5 meals per day; (2) Meal intervals not exceeding 4 h; (3) Adequate dietary variety; (4) Daily consumption of fruit and vegetables in at least 2–3 meals; and (5) Daily consumption of milk and dairy products in at least 2–3 meals. Each criterion was scored dichotomously as 1 point (“yes”) or 0 points (“no”), resulting in a total score ranging from 0 to 5 points, with higher scores indicating better adherence to the assessed dietary recommendations. Based on the total score, food records were classified into three categories: poor (0–2 points), good (3–4 points), very good (5 points). The obtained scores were used to compare selected dietary behaviors and the overall quality of food records between recipients in the early and late post-transplant periods.

### 2.4. Immunosuppressive Therapy

Information regarding immunosuppressive therapy was obtained from medical records and verified during routine outpatient medical consultations. Data collected included the type of immunosuppressive regimen, the number of immunosuppressive agents used, the specific immunosuppressive drugs administered, and their current daily doses. The analyzed immunosuppressive agents were classified into three major categories: calcineurin inhibitors (tacrolimus and cyclosporine), antiproliferative agents (mycophenolate mofetil, mycophenolic acid, and azathioprine), and corticosteroids. In addition, tacrolimus and cyclosporine formulations were recorded separately, including Prograf^®^, Advagraf^®^, Envarsus^®^, Equoral^®^, and Neoral^®^. Corticosteroid therapy included prednisone (Encorton^®^), methylprednisolone (Metypred^®^), and deflazacort (Calcort^®^). The selection of specific immunosuppressive formulations reflected routine clinical practice at the study center and was made by the treating transplant physician based on individual patient characteristics, clinical considerations (including treatment adherence and dosing convenience when appropriate), and the availability of reimbursed medications within the Polish healthcare system. The choice of commercial formulations was not related to the study protocol. Patients were classified according to the type of immunosuppressive regimen, including tacrolimus-based and cyclosporine-based therapy, the use of antiproliferative agents, corticosteroid treatment, and the presence of triple immunosuppressive therapy consisting of a calcineurin inhibitor, an antiproliferative agent, and a corticosteroid. Daily doses of mycophenolate preparations and corticosteroids were recorded for analysis. Corticosteroid doses were expressed as prednisone-equivalent doses. In addition, tacrolimus trough concentrations (C_0_) obtained during routine laboratory monitoring were collected and included in the analysis.

### 2.5. Statistical Analysis

Statistical analyses were performed using Statistica software (version 13.3; TIBCO Software Inc., Palo Alto, CA, USA). The normal distribution of continuous variables was assessed using the Shapiro–Wilk test. As several variables did not meet the assumptions of normality and the study sample was relatively small, non-parametric statistical methods were applied for group comparisons. Continuous variables are presented as mean ± standard deviation (SD), together with median values and ranges to facilitate interpretation of the data distribution. Categorical variables are presented as absolute numbers and percentages. Comparisons between kidney transplant recipients in the early (≤1 year) and late (>1 year) post-transplant periods were performed using the Mann–Whitney U test for continuous variables and the chi-square test for categorical variables. Fisher’s exact test was used when the expected cell count was less than five. The Mann–Whitney U test was applied to compare anthropometric measurements, body composition parameters obtained by bioelectrical impedance analysis (BIA), laboratory measurements, and dietary variables assessed using the FFQ-6 questionnaire. Nutritional risk categories identified using the NRS-2002 questionnaire and other categorical variables were compared using chi-square or Fisher’s exact tests, as appropriate. Missing data were handled using available-case analysis; therefore, the number of observations may differ between variables depending on data availability. All statistical tests were two-tailed, and a *p*-value < 0.05 was considered statistically significant. No adjustment for multiple comparisons was performed. In addition, no multivariable analyses were conducted because reliable adjustment for multiple potential confounding variables was not considered statistically appropriate in this relatively small dataset and could have resulted in overfitted models and unstable estimates.

### 2.6. Ethical Considerations

The study was conducted in accordance with the principles of the Declaration of Helsinki. The study protocol entitled “Immunosuppressive treatment regimen and nutritional disorders in kidney transplant recipients” was approved by the Bioethics Committee of the Medical University of Warsaw (approval No. AKBE/252/2022) on 7 November 2022. Permission to conduct the study at the Transplant Medicine and Nephrology Clinic in Warsaw and to access patients’ medical records for scientific purposes was granted by the Head of the Clinic. All participants provided written informed consent prior to enrollment in the study. Participation was voluntary, and all collected data were anonymized before statistical analysis.

## 3. Results

### 3.1. Characteristics of Study Population

The study included 38 kidney transplant recipients, comprising 15 patients in the early post-transplant group (≤1 year after transplantation) and 23 patients in the late post-transplant group (>1 year after transplantation). The sociodemographic and clinical characteristics of the study population are presented in Table 1 and Table 2.

No statistically significant differences were observed between the early and late post-transplant groups with respect to sex, age, place of residence, educational attainment, or household income (Table 1). Men predominated in the early post-transplant group (66.7%), whereas women were more frequent in the late post-transplant group (56.5%). The mean age was 44.9 ± 14.0 years in the early post-transplant group and 53.5 ± 14.1 years in the late post-transplant group. Most participants in both groups resided in urban areas. Secondary education was the most common level in the early post-transplant group, whereas secondary and higher education were equally represented in the late post-transplant group. Most participants reported lower-middle, upper-middle, or high household income, and no statistically significant differences in income distribution were identified between the groups.

Time since transplantation differed significantly between the groups (*p* < 0.0001) (Table 2). Most recipients in both groups had undergone dialysis before transplantation, whereas pre-emptive transplantation was relatively uncommon. Similarly, deceased donor transplantation predominated in both groups, and no statistically significant differences were observed with respect to donor type or living-related transplantation. The prevalence of comorbidities was high in both groups, with hypertension being the most common condition, followed by lipid disorders and diabetes mellitus. No statistically significant differences in the prevalence of individual comorbidities were observed between the groups. Likewise, the duration of dialysis before transplantation did not differ significantly between the groups.

### 3.2. Nutritional Status Differences

#### 3.2.1. Anthropometric Measurements

The results of anthropometric measurements are presented in Table 3.

Anthropometric measurements were available for all participants with respect to body weight, height, and BMI, whereas selected circumference measurements were available for a smaller number of recipients in the late post-transplant group. No statistically significant differences were observed between the early and late post-transplant groups for any of the analyzed anthropometric parameters. Mean body weight and BMI did not differ significantly between groups, with the average BMI corresponding to the overweight category in both early (25.72 ± 5.28 kg/m^2^) and late (26.35 ± 5.96 kg/m^2^) post-transplant recipients. Likewise, measures of central adiposity, including waist circumference, hip circumference, and waist-to-hip ratio (WHR), did not differ significantly between the groups. Peripheral anthropometric measurements, including calf circumference and mid-upper arm circumference, also showed no statistically significant differences.

#### 3.2.2. Laboratory Measurements

The laboratory measurements of kidney transplant recipients according to post-transplant group are presented in Table 4.

For most laboratory parameters, no statistically significant differences were detected between recipients within the first year after transplantation and those transplanted more than one year post-transplantation. This applied to CRP, creatinine, total cholesterol, LDL cholesterol, HDL cholesterol, triglycerides, uric acid, sodium, potassium, calcium, iron, and serum 25(OH)D concentrations (all *p* > 0.05). Serum 25(OH)D concentrations were available only for four and three recipients, respectively. Therefore, although no statistically significant difference was observed, these findings should be interpreted with caution. Iron concentrations were available for only one recipient in the early post-transplant group and three recipients in the late post-transplant group. Therefore, statistical comparison was not performed.

Statistically significant differences were identified for three laboratory variables. Tacrolimus trough concentrations were significantly higher in the first-year group (11.20 ± 5.23 vs. 4.56 ± 1.07 ng/mL, *p* = 0.0001). These findings were based on measurements available for 15 and 21 recipients, respectively. Recipients within the first year after transplantation had significantly higher non-HDL cholesterol concentrations than recipients transplanted more than one year post-transplantation (153.29 ± 37.50 vs. 111.13 ± 50.84 mg/dL, *p* = 0.040). However, these results were based on measurements available for seven and eight recipients, respectively, and should therefore be interpreted with caution. Serum magnesium concentrations were significantly lower (1.64 ± 0.13 vs. 1.94 ± 0.15 mg/dL, *p* = 0.009). These findings were based on six and five available measurements, respectively, and should therefore be interpreted with caution.

#### 3.2.3. Body Composition

The results of bioelectrical impedance analysis (BIA) are presented in Table 5. As with the laboratory measurements, the number of available observations varied across variables because not all body composition parameters were available for every participant.

No statistically significant differences were detected between recipients within the first year after transplantation and those transplanted more than one year post-transplantation for any of the assessed body composition parameters (all *p* > 0.05). Specifically, fat mass (kg and %), skeletal muscle mass, fat-free mass (kg and %), total body water, intracellular and extracellular water compartments, ECW/ICW ratio, body cell mass, extracellular mass, protein mass, and mineral content did not differ significantly between the study groups.

#### 3.2.4. Nutritional Risk Screening (NRS-2002)

The results of nutritional risk screening according to the NRS-2002 questionnaire are presented in Table 6.

The majority of recipients in both the early and late post-transplant groups were classified as being at no nutritional risk according to the NRS-2002. Nutritional risk was identified in a minority of participants, affecting 13.3% of early post-transplant recipients and 34.8% of late post-transplant recipients. No statistically significant differences were found between the groups.

### 3.3. Dietary Intake Differences

#### 3.3.1. FFQ-6

The frequency of consumption of selected food products assessed using the FFQ-6 questionnaire is presented in Table 7.

No statistically significant differences were observed between the groups for the majority of the analyzed food products. The frequency of consumption of dairy products, fruit, vegetables, cereal products, legumes, meat, fish, and most beverages did not differ significantly between early and late post-transplant groups. The only statistically significant difference was observed for the frequency of adding sugar to beverages, which was higher among early post-transplant recipients than among late recipients (4.40 ± 2.10 vs. 3.09 ± 2.04, *p* = 0.048). No statistically significant differences were observed for the remaining food products assessed.

#### 3.3.2. 3-Day Dietary Record

The evaluation of 3-day dietary records according to post-transplant period is presented in Table 8.

Dietary record data were available for nine recipients in the early post-transplant group and ten recipients in the late post-transplant group. No statistically significant differences were observed between the groups in the overall dietary record assessment or any of the analyzed dietary habits. The majority of dietary records were classified as good or very good. Likewise, not statistically significant between-group differences were observed in adherence to any of the assessed dietary recommendations, including meal frequency, meal intervals, dietary variety, and consumption of fruit, vegetables, and dairy products.

### 3.4. Immunosuppressive Therapy Comparison

The comparison of immunosuppressive therapy between the early and late post-transplant groups is presented in Table 9.

Triple immunosuppressive therapy was the predominant treatment regimen in both groups and was administered to all recipients in the early post-transplant group and to the majority of recipients in the late post-transplant group. Tacrolimus-based regimens were the most commonly prescribed calcineurin inhibitor therapy in both groups, whereas cyclosporine-based regimens were used only in two recipients in the late post-transplant group. Similarly, mycophenolate-based therapy and corticosteroid treatment were administered to nearly all participants. Among tacrolimus formulations, Prograf^®^ was the most frequently prescribed preparation in both groups, whereas Advagraf^®^ was more commonly used in the late post-transplant group. Encorton^®^ was the predominant corticosteroid formulation, while Metypred^®^ and Calcort^®^ were prescribed only in a small number of late post-transplant recipients. Recipients in the early post-transplant group had significantly higher tacrolimus trough concentrations, prednisone-equivalent doses, and daily mycophenolate doses than recipients in the late post-transplant group. These differences were statistically significant (*p* = 0.001, *p* = 0.004, and *p* = 0.002, respectively). Detailed data are presented in Panel B of Table 9.

## 4. Discussion

### 4.1. Principal Findings

In this exploratory cross-sectional pilot study, most measures of nutritional status, body composition, dietary intake, and nutritional risk did not differ significantly between the early and late post-transplant groups. The main differences concerned immunosuppressive therapy and selected biochemical parameters. Recipients in the early post-transplant period had higher tacrolimus trough concentrations and higher prednisone-equivalent and mycophenolate doses, as well as lower serum magnesium and higher non-HDL cholesterol concentrations. Dietary habits did not differ significantly, although early post-transplant recipients reported more frequent addition of sugar to beverages. No significant differences were observed in anthropometric measurements, body composition, or nutritional risk.

In this clinically stable cohort, the observed differences were primarily related to immunosuppressive exposure and selected biochemical parameters rather than to nutritional status or dietary intake. The absence of significant differences in most nutritional measures should, however, be interpreted in the context of the small sample size and exploratory design. The findings therefore provide preliminary information that may help characterize differences across post-transplant periods, but they do not establish longitudinal changes or causal relationships.

### 4.2. Nutritional Status and Dietary Intake

No statistically significant differences were observed between the early and late post-transplant groups in body weight, BMI, anthropometric measurements, BIA-derived body composition, nutritional risk assessed using the NRS-2002, or most laboratory parameters. This finding is notable given that the first post-transplant year is commonly associated with weight gain and changes in body composition [5,11,12,21]. Previous studies have also reported considerable variability in nutritional outcomes after kidney transplantation, with body composition influenced by multiple clinical and treatment-related factors [5,8,15,22,23,24]. The absence of significant differences in the assessed nutritional measures in our cohort should therefore be considered in the context of this variability.

The findings for body composition and nutritional risk are also relevant to the interpretation of nutritional status after transplantation. BMI and conventional anthropometric measures do not distinguish between fat mass and lean tissue, while BIA provides additional information on body composition but may be affected by hydration status and other patient-related factors. Previous research has highlighted the heterogeneous nature of body composition changes after kidney transplantation and the value of using complementary assessment methods [5,23,25,26,27]. In the present study, no significant differences were identified across anthropometric, BIA, and NRS-2002 measures, indicating that no clear differences in the assessed nutritional parameters were identified according to post-transplant period.

Most FFQ-6 measures also did not differ significantly, with the only statistically significant finding being more frequent addition of sugar to beverages among early post-transplant recipients. This finding should be considered in the context of the metabolic changes and immunosuppressive treatment commonly observed during the early post-transplant period [6,15,28]. However, given the exploratory design and the number of dietary variables assessed, this isolated finding should be interpreted cautiously and should not be considered evidence of a broader difference in dietary quality according to post-transplant period. Previous studies have reported variability in dietary habits among kidney transplant recipients [5,10,13], supporting the need to interpret individual dietary findings within the broader nutritional context.

The 3-day dietary records provided a complementary assessment of selected dietary behaviors. No statistically significant differences were observed in the overall dietary record assessment or in the individual dietary recommendations assessed. Overall, the findings from both dietary assessment methods did not identify major differences in the assessed dietary parameters according to post-transplant period.

### 4.3. Immunosuppressive Therapy and Metabolic Implications

In the present study, the main differences between the post-transplant groups concerned immunosuppressive therapy, with higher tacrolimus trough concentrations, prednisone-equivalent doses, and mycophenolate doses observed in recipients within the first year after transplantation. Such treatment strategies are consistent with current recommendations for immunosuppressive management after kidney transplantation [7,8,16]. Immunosuppressive therapy is an important component of post-transplant care and may influence several metabolic parameters [15,16].

Among the metabolic findings, early post-transplant recipients had lower serum magnesium concentrations and higher non-HDL cholesterol concentrations. The lower magnesium concentrations observed in this group are consistent with the known effect of tacrolimus on renal magnesium handling. Calcineurin inhibitors can increase renal magnesium wasting by impairing tubular magnesium reabsorption, and tacrolimus exposure has been associated with lower serum magnesium concentrations in kidney transplant recipients [28,29]. Calcineurin inhibitors and corticosteroids may also contribute to dyslipidemia through effects on lipid metabolism [15,16]. This provides a potential context for the higher non-HDL cholesterol concentrations observed in the early post-transplant group.

Importantly, the differences in immunosuppressive therapy and selected biochemical parameters were not accompanied by statistically significant differences in nutritional status, body composition, dietary intake, or nutritional risk. Nutritional status is influenced by multiple factors, including dietary habits, physical activity, graft function, comorbidities, and immunosuppressive treatment, which should be considered when interpreting nutritional findings after transplantation.

An important contribution of the present study is the integrated assessment of immunosuppressive therapy, nutritional status, dietary intake, body composition, nutritional risk, and biochemical parameters within the same cohort of kidney transplant recipients. Previous studies have examined individual nutritional and metabolic outcomes in relation to immunosuppressive therapy after kidney transplantation, including diabetes, lipid abnormalities, body weight, body composition, and electrolyte disturbances [30]. In the present study, differences in immunosuppressive therapy were accompanied by differences in selected biochemical parameters, particularly tacrolimus exposure, serum magnesium, and non-HDL cholesterol concentrations, but not by statistically significant differences in nutritional status, body composition, dietary intake, or nutritional risk. The integrated assessment provides preliminary insight into the relationship between immunosuppressive therapy, metabolic parameters and nutritional status in kidney transplant recipients. Given the pilot nature of the study, these findings require confirmation in larger prospective cohorts.

### 4.4. Strengths and Limitations

The present study has several strengths. First, it was conducted within an ongoing prospective cohort of kidney transplant recipients. Although the present analysis is cross-sectional, the prospective design provides a valuable framework for future investigations and enhances the overall scientific value of the project.

The study also has several limitations that should be considered when interpreting the findings. First, the relatively small sample size limits the statistical power of the analyses, particularly after stratification according to post-transplant period. Therefore, the findings should be considered preliminary and interpreted with caution. Multivariable analyses were not performed because of the limited sample size, and the potential influence of unmeasured or residual confounding cannot be excluded. Second, the cross-sectional design does not allow assessment of temporal or causal relationships between immunosuppressive therapy, nutritional status, dietary intake, body composition, and metabolic parameters. In addition, the predefined one-year cutoff does not capture the continuous nature of post-transplant changes.

Some laboratory and questionnaire-based variables were available only for a subset of participants, resulting in different numbers of observations across analyses. In addition, dietary assessment was based on self-reported FFQ-6 questionnaires and 3-day dietary records and did not include quantitative assessment of energy and individual nutrient intake. Body composition was assessed using BIA, which may be influenced by hydration status despite standardized measurement procedures. Finally, the study was conducted at a single transplant center and included clinically stable recipients receiving routine outpatient care, which may limit the generalizability of the findings to other transplant populations and settings.

Despite these limitations, the study provides a comprehensive evaluation of nutritional status, dietary intake, body composition, biochemical parameters, and immunosuppressive therapy within the same cohort of kidney transplant recipients. This integrated approach offers a broader perspective on nutritional and metabolic health after kidney transplantation and provides a valuable foundation for future prospective analyses.

## 5. Conclusions

In this pilot study, the observed differences between the early and late post-transplant groups concerned primarily immunosuppressive exposure and selected metabolic parameters, whereas no statistically significant differences were identified for most measures of nutritional status or dietary intake. However, given the small sample size and the exploratory nature of this pilot study, these findings should be considered preliminary and hypothesis-generating rather than confirmatory. The results also support the value of comprehensive assessment, combining anthropometric measurements, body composition analysis, dietary evaluation, nutritional screening, and laboratory parameters, to characterize the nutritional and metabolic profile of kidney transplant recipients. Larger prospective multicenter studies involving more diverse kidney transplant populations are needed to validate these observations and establish their broader generalizability.

## Figures and Tables

**Table 1:** Sociodemographic characteristics of kidney transplant recipients according to post-transplant period.

Variable	Early Post-Transplant (≤1 Year) (*n* = 15)	Late Post-Transplant (>1 Year) (*n* = 23)	*p*-Value
Female sex, *n* (%)	5 (33.3)	13 (56.5)	NS
Age, years; mean ± SD, range (median)	44.9 ± 14.0; 19–64 (49)	53.5 ± 14.1; 24–77 (55)	NS
Place of residence, *n* (%) ^1^			NS
Rural area	4 (26.7)	7 (30.4)	
Small town	2 (13.3)	2 (8.7)	
Medium-sized city	4 (26.7)	6 (26.1)	
Large city	5 (33.3)	8 (34.8)	
Educational level, *n* (%)			NS
Primary	1 (7.1)	2 (9.1)	
Vocational	3 (21.4)	6 (27.3)	
Secondary	6 (42.9)	7 (31.8)	
Higher	4 (28.6)	7 (31.8)	
Monthly household income, *n* (%) ^2^			NS
Low income	0 (0.0)	1 (4.8)	
Lower-middle income	6 (46.2)	6 (28.6)	
Upper-middle income	3 (23.1)	6 (28.6)	
High income	4 (30.8)	8 (38.1)	

^1^ Place of residence categories were defined as follows: rural area, small town (<20,000 inhabitants), medium-sized city (20,000–100,000 inhabitants), and large city (>100,000 inhabitants). ^2^ Income categories were defined as follows: low income (<1000 PLN/month per household member), lower-middle income (1000–2500 PLN/month), upper-middle income (2501–4000 PLN/month), and high income (>4000 PLN/month). Abbreviations: NS, not significant.

**Table 2:** Clinical characteristics of kidney transplant recipients according to post-transplant period.

Variable	Early Post-Transplant (≤1 Year) (*n* = 15)	Late Post-Transplant (>1 Year) (*n* = 23)	*p*-Value
Time since transplantation, days; mean ± SD; range (median)	147.27 ± 127.86; 10–356 (142)	2616.74 ± 2176.25; 434–7948 (1731)	<0.0001
Dialysis before transplantation, *n* (%)	14 (93.3)	16 (76.2)	NS
Pre-emptive transplantation, *n* (%)	1 (6.7)	5 (23.8)	NS
Donor type, *n* (%)			NS
—Living donor	6 (42.9)	5 (23.8)	
—Deceased donor	8 (57.1)	16 (76.2)	
Living-related transplantation, *n* (%)			NS
—Yes	5 (35.7)	5 (23.8)	
—No	9 (64.3)	16 (76.2)	
Presence of comorbidities, *n* (%)	13 (86.7)	22 (95.7)	NS
Hypertension, *n* (%)	12 (80.0)	21 (91.3)	NS
Diabetes mellitus, *n* (%)	4 (26.7)	8 (34.8)	NS
Lipid disorders, *n* (%)	5 (33.3)	13 (56.5)	NS
Dialysis duration before transplantation, months; mean ± SD; range (median)	27.87 ± 22.02; 0–60 (24)	16.35 ± 14.25; 0–48 (13)	NS

Abbreviations: NS, not significant.

**Table 3:** Anthropometric measurements of kidney transplant recipients according to post-transplant period.

Variable	Early Post-Transplant (≤1 Year), (*n* = 15)	Late Post-Transplant (>1 Year), (*n* = 23)	*p*-Value
	*n*; Mean ± SD; Range (Median)	*n*; Mean ± SD; Range (Median)	
Body weight, kg	15; 77.82 ± 16.99; 51–105 (77.5)	23; 76.69 ± 19.80; 46–115 (75)	NS
Height, cm	15; 173.80 ± 6.42; 162–187 (176)	23; 170.26 ± 12.28; 150–194 (168)	NS
BMI, kg/m^2^	15; 25.72 ± 5.28; 18.29–35.40 (27.46)	23; 26.35 ± 5.96; 16.00–37.20 (24.31)	NS
Waist circumference, cm	15; 91.87 ± 17.72; 64–120 (90)	19; 95.21 ± 21.34; 64–160 (93)	NS
Hip circumference, cm	15; 98.67 ± 16.66; 50–123 (99)	19; 102.32 ± 10.83; 78–121 (102)	NS
Waist-to-hip ratio (WHR)	15; 0.94 ± 0.15; 0.77–1.28 (0.94)	20; 0.94 ± 0.18; 0.70–1.45 (0.91)	NS
Right calf circumference, cm	15; 37.13 ± 3.70; 31–43 (37)	20; 38.40 ± 3.50; 30–46 (38.5)	NS
Left calf circumference, cm	15; 37.43 ± 3.39; 31–43 (39)	20; 38.55 ± 3.75; 30–45 (38.5)	NS
Mid-upper arm circumference, cm	15; 29.97 ± 4.68; 22.5–38 (31)	19; 30.16 ± 3.82; 24–37 (30)	NS

Abbreviations: BMI, body mass index; WHR, waist-to-hip ratio; NS, not significant.

**Table 4:** Laboratory measurements of kidney transplant recipients according to post-transplant period.

Variable	Early Post-Transplant (≤1 Year)	Late Post-Transplant (>1 Year)	*p*-Value
	*n*; Mean ± SD; Range (Median)	*n*; Mean ± SD; Range (Median)	
CRP, mg/L	15; 4.75 ± 10.28; 0.60–37.90 (0.80)	23; 4.42 ± 6.65; 0.60–25.00 (1.70)	NS
Total cholesterol, mg/dL	8; 203.25 ± 49.58; 142–287 (200)	9; 167.44 ± 49.09; 99–273 (164)	NS
HDL cholesterol, mg/dL	8; 58.50 ± 14.24; 40–82 (61)	8; 54.63 ± 13.96; 40–78 (49)	NS
LDL cholesterol, mg/dL	8; 106.13 ± 42.92; 42–177 (96.5)	8; 81.88 ± 49.55; 18–186 (72.5)	NS
Non-HDL cholesterol, mg/dL	7; 153.29 ± 37.50; 102–205 (146)	8; 111.13 ± 50.84; 56–227 (102)	0.040
Triglycerides, mg/dL	8; 205.00 ± 60.58; 126–300 (218.5)	11; 158.45 ± 91.73; 80–393 (139)	NS
Uric acid, mg/dL	13; 6.38 ± 1.50; 3.50–8.40 (6.40)	21; 5.86 ± 1.42; 3.10–9.60 (6.10)	NS
Creatinine, mg/dL	15; 1.48 ± 0.35; 0.92–2.09 (1.41)	23; 1.66 ± 0.86; 0.81–4.79 (1.55)	NS
Tacrolimus concentration, ng/mL	15; 11.20 ± 5.23; 2.80–19.60 (12.0)	21; 4.56 ± 1.07; 2.50–6.70 (4.30)	0.0001
Magnesium, mg/dL	6; 1.64 ± 0.13; 1.53–1.86 (1.59)	5; 1.94 ± 0.15; 1.77–2.14 (1.87)	0.009
Potassium, mmol/L	15; 4.62 ± 0.46; 4.00–5.47 (4.54)	23; 4.38 ± 0.50; 3.61–5.49 (4.32)	NS
Sodium, mmol/L	15; 140.31 ± 2.22; 136.10–143.60 (141.30)	22; 141.05 ± 2.56; 133.70–144.30 (141.65)	NS
Calcium, mg/dL	11; 10.56 ± 0.92; 9.70–12.50 (10.20)	6; 9.87 ± 0.20; 9.70–10.20 (9.80)	NS
Iron, µg/dL	1; 106.00 ± 0.00; 106–106 (106)	3; 131.00 ± 36.59; 93–166 (134)	NS
Vitamin D_3_, ng/mL	4; 29.17 ± 11.12; 20.40–44.65 (25.81)	3; 36.05 ± 2.05; 33.93–38.02 (36.21)	NS

Abbreviations: CRP, *C*-reactive protein; HDL, high-density lipoprotein; LDL, low-density lipoprotein; NS, not significant.

**Table 5:** Body composition parameters of kidney transplant recipients according to post-transplant period.

Variable	Early Post-Transplant (≤1 Year)	Late Post-Transplant (>1 Year)	*p*-Value
	*n*; Mean ± SD; Range (Median)	*n*; Mean ± SD; Range (Median)	
Fat mass (FAT), kg	14; 23.28 ± 12.94; 7.43–43.62 (19.80)	21; 24.87 ± 10.88; 7.10–44.96 (22.91)	NS
Body fat (FAT), %	13; 29.09 ± 11.58; 13.04–49.36 (26.94)	21; 31.87 ± 10.10; 12.03–49.36 (31.38)	NS
Skeletal muscle mass, kg	13; 25.57 ± 5.90; 17.33–33.76 (28.13)	21; 24.79 ± 7.50; 13.84–41.78 (21.79)	NS
Fat-free mass (FFM), kg	14; 51.95 ± 12.26; 20.69–66.96 (56.23)	20; 52.98 ± 13.25; 31.91–85.09 (50.38)	NS
Fat-free mass (FFM), %	13; 70.91 ± 11.58; 50.64–86.96 (73.06)	20; 69.10 ± 9.79; 50.64–87.97 (69.84)	NS
Total body water (TBW), %	13; 54.63 ± 12.67; 39.16–90.00 (54.98)	21; 50.64 ± 6.15; 39.16–61.34 (49.41)	NS
Intracellular water (ICW), %	14; 51.97 ± 10.87; 20.00–61.00 (55.69)	21; 54.60 ± 2.54; 50.96–59.37 (54.22)	NS
Extracellular water (ECW), %	14; 48.03 ± 10.88; 39.00–80.00 (44.31)	21; 45.52 ± 2.69; 40.62–49.96 (45.77)	NS
ECW/ICW ratio	14; 1.09 ± 0.88; 0.639–4.00 (0.80)	21; 0.83 ± 0.08; 0.68–0.96 (0.84)	NS
Body cell mass (BCM), kg	14; 28.76 ± 6.08; 19.43–36.17 (31.38)	21; 28.52 ± 7.46; 16.59–47.06 (27.07)	NS
Extracellular mass (ECM), kg	14; 24.62 ± 6.63; 6.93–34.99 (25.88)	21; 24.15 ± 5.64; 15.32–38.03 (23.18)	NS
Protein mass (Prot), kg	14; 10.16 ± 2.93; 2.51–14.34 (10.66)	20; 9.76 ± 3.02; 3.71–16.07 (9.41)	NS
Mineral content (Mnrl), kg	14; 3.66 ± 1.16; 0.15–5.03 (4.02)	20; 3.74 ± 0.81; 2.61–5.64 (3.66)	NS

Abbreviations: FAT, fat mass; FFM, fat-free mass; TBW, total body water; ICW, intracellular water; ECW, extracellular water; BCM, body cell mass; ECM, extracellular mass; NS, not significant.

**Table 6:** Nutritional Risk Screening (NRS-2002) results according to post-transplant period.

Variable	Early Post-Transplant (≤1 Year) (*n* = 15)	Late Post-Transplant (>1 Year) (*n* = 23)	*p*-Value
No nutritional risk, *n* (%)	13 (86.67)	15 (65.22)	NS
Nutritional risk present, n (%)	2 (13.33)	8 (34.78)	NS

Abbreviations: NS, not significant.

**Table 7:** Frequency of consumption of selected food products according to post-transplant period (FFQ-6).

Food Product	Early Post-Transplant (≤1 Year)	Late Post-Transplant (>1 Year)	*p*-Value
	Mean ± SD; Range (Median)	Mean ± SD; Range (Median)	
Sugar added to beverages	4.40 ± 2.10; 1–6 (6)	3.09 ± 2.04; 1–6 (3)	0.048
Milk and natural dairy beverages	3.67 ± 1.59; 1–6 (4)	3.78 ± 1.31; 1–6 (4)	NS
Sweetened dairy beverages	2.33 ± 1.29; 1–4 (2)	1.91 ± 1.20; 1–4 (1)	NS
Cheese	2.87 ± 1.55; 1–5 (3)	2.61 ± 1.41; 1–5 (3)	NS
Eggs	3.73 ± 1.10; 1–6 (4)	3.39 ± 0.99; 1–5 (4)	NS
Fruit	4.47 ± 1.06; 2–6 (5)	4.26 ± 1.01; 2–6 (4)	NS
Apples and pears	4.07 ± 1.10; 1–5 (4)	3.78 ± 1.13; 1–5 (4)	NS
Bananas	3.13 ± 1.36; 1–5 (3)	3.04 ± 1.36; 1–5 (3)	NS
Avocado	1.20 ± 0.56; 1–3 (1)	1.78 ± 1.09; 1–4 (1)	NS
Vegetables	4.47 ± 0.52; 4–5 (5)	4.09 ± 0.73; 3–5 (4)	NS
Carrots and peppers	4.07 ± 0.80; 2–5 (4)	3.87 ± 0.69; 2–5 (4)	NS
Potatoes	3.93 ± 0.70; 3–5 (4)	3.52 ± 1.16; 1–5 (4)	NS
Legumes	1.93 ± 0.80; 1–4 (2)	2.13 ± 0.92; 1–4 (2)	NS
Wholegrain bread	4.07 ± 1.39; 1–6 (4)	3.91 ± 1.68; 1–6 (5)	NS
White bread	3.47 ± 1.73; 1–6 (4)	3.96 ± 1.58; 1–6 (5)	NS
Butter	4.07 ± 1.33; 1–5 (5)	4.52 ± 1.53; 1–6 (5)	NS
Sausages	2.87 ± 1.55; 1–6 (3)	2.61 ± 1.20; 1–4 (2)	NS
Red meat	3.00 ± 0.93; 1–4 (3)	3.00 ± 1.28; 1–5 (3)	NS
Poultry and rabbit meat	3.00 ± 0.93; 1–4 (3)	3.22 ± 1.13; 1–5 (3)	NS
Lean fish	2.60 ± 0.74; 1–4 (3)	2.26 ± 0.92; 1–4 (2)	NS
Fatty fish	2.53 ± 0.99; 1–4 (2)	1.96 ± 0.93; 1–4 (2)	NS
Fruit juices	2.80 ± 1.70; 1–6 (3)	1.96 ± 1.19; 1–5 (2)	NS
Vegetable juices	1.87 ± 1.13; 1–5 (2)	1.70 ± 0.82; 1–3 (1)	NS

Higher scores indicate more frequent consumption according to the FFQ-6 scale (1 = never/almost never; 6 = several times daily). *p*-values were calculated using the Mann–Whitney U test. Abbreviations: NS, not significant.

**Table 8:** Evaluation of the 3-day dietary records according to post-transplant period.

Variable	Early Post-Transplant (≤1 Year)	Late Post-Transplant (>1 Year)	*p*-Value
Overall dietary record assessment, *n* (%)			NS
—Poor	0 (0.0)	4 (40.0)	
—Good	8 (88.9)	6 (60.0)	
—Very good	1 (11.1)	0 (0.0)	
4–5 meals per day, *n* (%)			NS
—Yes	8 (88.9)	7 (70.0)	
—No	1 (11.1)	3 (30.0)	
Meal intervals ≤ 4 h, *n* (%)			NS
—Yes	8 (88.9)	5 (50.0)	
—No	1 (11.1)	5 (50.0)	
Dietary variety, *n* (%)			NS
—Yes	8 (88.9)	4 (40.0)	
—No	1 (11.1)	6 (60.0)	
Fruit and vegetables consumed daily in 2–3 meals, *n* (%)			NS
—Yes	7 (77.8)	8 (80.0)	
—No	2 (22.2)	2 (20.0)	
Milk and dairy products consumed daily in 2–3 meals, *n* (%)			NS
—Yes	2 (22.2)	1 (10.0)	
—No	7 (77.8)	9 (90.0)	

Data are presented as numbers (%). *p*-values were calculated using Fisher’s exact test. Abbreviations: NS, not significant.

**Table 9:** Comparison of immunosuppressive therapy according to post-transplant period. (**A**). Type of immunosuppressive regimen. (**B**). Daily doses of major immunosuppressive agents.

**(A)**			
**Variable**	**Early Post-Transplant (≤1 Year)** **(*n* = 15)**	**Late Post-Transplant (>1 Year)** **(*n* = 23)**	
Triple immunosuppressive therapy, *n* (%)	15 (100.0)	21 (91.3)	
Tacrolimus-based regimen, *n* (%)	15 (100.0)	21 (91.3)	
Cyclosporine-based regimen, *n* (%)	0 (0.0)	2 (8.7)	
Mycophenolate-based therapy, *n* (%)	15 (100.0)	21 (91.3)	
Corticosteroid therapy, *n* (%)	15 (100.0)	22 (95.7)	
Tacrolimus formulations, *n* (%)			
—Prograf^®^	9 (60.0)	11 (47.8)	
—Envarsus^®^	4 (26.7)	2 (8.7)	
—Advagraf^®^	2 (13.3)	8 (34.8)	
Cyclosporine formulations, *n* (%)			
—Equoral^®^	0 (0.0)	1 (4.3)	
—Neoral^®^	0 (0.0)	1 (4.3)	
Corticosteroid formulations, *n* (%)			
—Encorton^®^	15 (100.0)	18 (73.1)	
—Metypred^®^	0 (0.0)	3 (13.0)	
—Calcort^®^	0 (0.0)	1 (4.3)	
**(B)**			
**Variable**	**Early Post-Transplant (≤1 year)**	**Late Post-Transplant (>1 year)**	***p*** **-Value**
Tacrolimus trough concentration, ng/mL, median (range)	6.0 (1.0–17.0)	2.5 (0.75–4.0)	0.001
Prednisone-equivalent dose, mg/day, median (range)	7.5 (4.75–20.0)	5.0 (4.0–7.5)	0.004
Mycophenolate dose, mg/day, median (range)	2000 (500–2000)	1000 (0.75–2000)	0.002

## Data Availability

The data supporting the findings of this study are available from the corresponding author upon reasonable request. Access to the data is restricted due to privacy concerns and ethical requirements.

## Supplementary Materials

The following supporting information can be downloaded at: https://www.mdpi.com/article/10.3390/nu18162725/s1, Comparison of immunosuppressive therapy according to post-transplant period, Supplementary Files S1–S5.

## Abbreviations

The following abbreviations are used in this manuscript:

BCM	Body Cell Mass
BIA	Bioelectrical Impedance Analysis
BMI	Body Mass Index
CRP	*C*-Reactive Protein
ECM	Extracellular Mass
ECW	Extracellular Water
ESPEN	European Society for Clinical Nutrition and Metabolism
FAT	Body Fat Mass
FFM	Fat-Free Mass
FFQ-6	Food Frequency Questionnaire (62-item version)
HDL-C	High-Density Lipoprotein Cholesterol
ICW	Intracellular Water
LDL-C	Low-Density Lipoprotein Cholesterol
Mnrl	Mineral Content
NRS-2002	Nutritional Risk Screening 2002
NS	Not Significant
Prot	Protein Mass
SD	Standard Deviation
STROBE	the Strengthening the Reporting of Observational Studies in Epidemiology Statement
TBW	Total Body Water
WHR	Waist-to-Hip Ratio
