# Supplementary material for: Nutritional Status, Dietary Intake and Immunosuppressive Therapy in Kidney Transplant Recipients: Differences Between Early and Late Post-Transplant Period—A Cross-Sectional Pilot Study
Source: Nutrients. 2026 Aug 20;18(16):2725. doi: 10.3390/nu18162725 (PMC13516085; doi:10.3390/nu18162725)
Supplement: Supplementary file 1 [file nutrients-18-02725-s001.zip › nutrients-4445511-supplementary.pdf]

Supplementary File S1

STROBE Statement—checklist of items that should be included in reports of observational studies

	Item No.	Recommendation	Page No.	Relevant text from manuscript
**Title and abstract**	1	(*a*) Indicate the study's design with a commonly used term in the title or the abstract		
		(*b*) Provide in the abstract an informative and balanced summary of what was done and what was found		
**Introduction**				
Background/rationale	2	Explain the scientific background and rationale for the investigation being reported		
Objectives	3	State specific objectives, including any prespecified hypotheses		
**Methods**				
Study design	4	Present key elements of study design early in the paper		
Setting	5	Describe the setting, locations, and relevant dates, including periods of recruitment, exposure, follow-up, and data collection		
Participants	6	(*a*) *Cohort study*—Give the eligibility criteria, and the sources and methods of selection of participants. Describe methods of follow-up<br>*Case-control study*—Give the eligibility criteria, and the sources and methods of case ascertainment and control selection. Give the rationale for the choice of cases and controls<br>*Cross-sectional study*—Give the eligibility criteria, and the sources and methods of selection of participants		
		(*b*) *Cohort study*—For matched studies, give matching criteria and number of exposed and unexposed<br>*Case-control study*—For matched studies, give matching criteria and the number of controls per case		
Variables	7	Clearly define all outcomes, exposures, predictors, potential confounders, and effect modifiers. Give diagnostic criteria, if applicable		
Data sources/ measurement	8*	For each variable of interest, give sources of data and details of methods of assessment (measurement). Describe comparability of assessment methods if there is more than one group		
Bias	9	Describe any efforts to address potential sources of bias		

Study size	10	Explain how the study size was arrived at

Continued on next page

Quantitative variables	11	Explain how quantitative variables were handled in the analyses. If applicable, describe which groupings were chosen and why
Statistical methods	12	(*a*) Describe all statistical methods, including those used to control for confounding
		(*b*) Describe any methods used to examine subgroups and interactions
		(*c*) Explain how missing data were addressed
		(*d*) *Cohort study*—If applicable, explain how loss to follow-up was addressed<br>*Case-control study*—If applicable, explain how matching of cases and controls was addressed<br>*Cross-sectional study*—If applicable, describe analytical methods taking account of sampling strategy
		(*e*) Describe any sensitivity analyses
**Results**		
Participants	13*	(a) Report numbers of individuals at each stage of study—eg numbers potentially eligible, examined for eligibility, confirmed eligible, included in the study, completing follow-up, and analysed
		(b) Give reasons for non-participation at each stage
		(c) Consider use of a flow diagram
Descriptive data	14*	(a) Give characteristics of study participants (eg demographic, clinical, social) and information on exposures and potential confounders
		(b) Indicate number of participants with missing data for each variable of interest
		(c) *Cohort study*—Summarise follow-up time (eg, average and total amount)
Outcome data	15*	*Cohort study*—Report numbers of outcome events or summary measures over time
		Case-control study—Report numbers in each exposure category, or summary measures of exposure
		Cross-sectional study—Report numbers of outcome events or summary measures
Main results	16	(*a*) Give unadjusted estimates and, if applicable, confounder-adjusted estimates and their precision (eg, 95% confidence interval). Make clear which confounders were adjusted for and why they were included
		(*b*) Report category boundaries when continuous variables were categorized
		(*c*) If relevant, consider translating estimates of relative risk into absolute risk for a meaningful time period

Continued on next page

Other analyses	17	Report other analyses done—eg analyses of subgroups and interactions, and sensitivity analyses
**Discussion**		
Key results	18	Summarise key results with reference to study objectives
Limitations	19	Discuss limitations of the study, taking into account sources of potential bias or imprecision. Discuss both direction and magnitude of any potential bias
Interpretation	20	Give a cautious overall interpretation of results considering objectives, limitations, multiplicity of analyses, results from similar studies, and other relevant evidence
Generalisability	21	Discuss the generalisability (external validity) of the study results
**Other information**		
Funding	22	Give the source of funding and the role of the funders for the present study and, if applicable, for the original study on which the present article is based

*Give information separately for cases and controls in case-control studies and, if applicable, for exposed and unexposed groups in cohort and cross-sectional studies.

Note: An Explanation and Elaboration article discusses each checklist item and gives methodological background and published examples of transparent reporting. The STROBE checklist is best used in conjunction with this article (freely available on the Web sites of PLoS Medicine at http://www.plosmedicine.org/, Annals of Internal Medicine at http://www.annals.org/, and Epidemiology at http://www.epidem.com/). Information on the STROBE Initiative is available at www.strobe-statement.org.

Supplementary File S2

……………………

Numer ID

„NOWY PRZESZCZEP"

KWESTIONARIUSZ

<u>I WIZYTA</u>

I. INFORMACJE WSTĘPNE

**Data:**	**Numer ID:**
**Inicjały dietetyka:**	**Inicjały pacjenta:**

Data kolejnej wizyty: ……………………….

II. DANE OSOBOWE, DEMOGRAFICZNE I EKONOMICZNE

Wiek:	
Tel. kontaktowy:	

PŁEĆ

☐ KOBIETA	☐ MĘŻCZYZNA

MIEJSCE ZAMIESZKANIA

Nazwa miasta: …………………………

☐ WIEŚ	☐ MIASTO MAŁE (poniżej 20 tys. mieszkańców)	☐ MIASTO ŚREDNIE (20-100 tys. mieszkańców)	☐ MIASTO DUŻE (100 tys. mieszkańców)

WYKSZTAŁCENIE

☐ PODSTAWOWE	☐ ŚREDNIE	☐ ZAWODOWE	☐ WYŻSZE

MIESIĘCZNY DOCHÓD NA CZŁONKA RODZINY (netto)

☐ pon. 1000 zł	☐ 1000-2500 zł	☐ 2501-4000 zł	☐ pow. 4000 zł

WYZNANIE

☐ KOŚCIÓŁ KATOLICKI	☐ KOŚCIÓŁ PRAWOSŁAWNY	☐ INNE	☐ NIE NALEŻĘ DO ŻADNEGO WYZNANIA

III. INFORMACJE DOTYCZĄCE PRZESZCZEPU

DATA PRZESZCZEPU:	

CZY PRZESZCZEP BYŁ OD ZMARŁEGO CZY OD ŻYWEGO DAWCY?

☐ ZMARŁY DAWCA	☐ ŻYWY DAWCA	☐ NIE WIADOMO

JAKA BYŁA PRZYCZYNA PRZESZCZEPU?

☐ NIEWYDOLNOŚĆ NEREK	☐ CUKRZYCA	☐ NADCIŚNIENIE
☐ ZAPALENIE KŁĘBUSZKÓW NEREK	☐ GENETYCZNA	☐ INNE

CZY BYŁ PAN/ PANI DIALIZOWANY/ DIALIZOWANA PRZED PRZESZCZEPEM?

☐ TAK	☐ NIE

Jeśli tak, to ile czasu: ……………………………………………………………

CZY PRZESZCZEP BYŁ OD CZŁONKA RODZINY?

☐ TAK	☐ NIE

IV. WYSTĘPOWANIE CHORÓB WSPÓŁISTNIEJĄCYCH

CHOROBY WSPÓŁISTNIEJĄCE

Czy występują u Pani/Pana jakieś choroby?

TAK*	NIE

*Jeśli tak, to uzupełniamy poniżej z dokumentacji medycznej

Lp.	NAZWA CHOROBY	KOD ICD
1.		
2.		
3.		
4.		
5.		
6.		
7.		

V. PRZYJMOWANE LEKI

**SCHEMAT IMMUNOSUPRESJI**		**INNE LEKI**
Nazwa leku	Dawkowanie	

VI. POMIARY ANTROPOMETRYCZNE

WAGA [kg]:	WZROST [cm]:

OBWÓD TALII [cm]:	OBWÓD BIODER [cm]:
OBWÓD ŁYDKI [cm]:	P:
	L:
OBWÓD RAMIENIA [cm]:	

VII. DIETA I UŻYWKI

Czy spożywa Pan/Pani fast-foody (typu hamburgery, pizza, hot-dogi)?

☐ TAK	☐ NIE

Jeśli tak, to jak często w miesiącu? ……………………………………………..

Czy zdarza się Panu/Pani jeść „na mieście" (typu restauracje, kawiarnie)?

☐ TAK	☐ NIE

Jeśli tak, to jak często w miesiącu? ………………………………………………..

Czy korzysta Pan/Pani z cateringu dietetycznego?

☐ TAK	☐ NIE

Jeśli tak, to jak często w miesiącu? ………………………………………………..

Czy pali Pan/Pani papierosy?

☐ TAK	☐ NIE

Jeśli tak, to ile [sztuk/dzień]? ..…………………………………………………..

Czy spożywa Pan/ Pani kawę?

☐ TAK	☐ NIE

Jeśli tak, to ile [filiżanek/dzień]? ..……………………………………………..

VIII. WYNIKI BADAŃ BIOCHEMICZNYCH
(*(z dokumentacji medycznej)*

Lp.	Nazwa
1.	ALBUMINY:
2.	PREALBUMINY:
3.	CHOLESTEROL CAŁKOWITY:
4.	CHOLESTEROL HDL:
5.	CHOLESTEROL NIE-HDL:
6.	CHOLESTEROL LDL:
7.	TRIGLICERYDY:
8.	GLUKOZA NA CZCZO:
9.	KWAS MOCZOWY:
10.	MOCZNIK:
11.	KREATYNINA
12.	eGFR:
13.	TACROLIMUS:
14.	Magnez (Mg) w surowicy:
15.	Potas (K) w surowicy:
16.	Sód (Na) w surowicy:
17.	Wapń całkowity w surowicy:
18.	Żelazo (Fe) :
19.	Witamina D_3:

20.	BIAŁKO CRP:
21.	TSH:
22.	PTH:
23.	INNE:

IX. BADANIE BIOIMPEDANCJI ELEKTRYCZNEJ (BIA)

Nr badania: ……

FAT [kg] =<br>FAT [%] =<br>Musc [kg] =<br>TBW [%] =<br>ECW [%] =<br>ICW [%] =<br>ECW/ICW =	Prot [kg] =<br>Mnrl [kg] =<br>BCM [kg] =<br>ECM [kg] =<br><br>TARGETS<br>• T. Fat % =<br>• T. Wgt kg =<br>• T. Wtr % =

X. OCENA 3-DNIOWEGO DZIENNICZKA SPOŻYCIA

☐ prześle drogą mailową	☐ przyniesie na kolejną wizytę	☐ nie ustalono

OCENA JAKOŚCIOWA DZIENNICZKA

1. Czy liczba posiłków wynosi 4-5 dziennie? TAK/NIE
2. Czy przerwy między posiłkami wynoszą nie więcej niż 4 h? TAK/NIE
3. Czy dieta pacjenta jest urozmaicona? TAK/NIE

4. Czy w diecie pacjenta owoce/ warzywa występują codziennie w 2-3 posiłkach? TAK/NIE
5. Czy w diecie pacjenta mleko i produkty mleczne występują codziennie w 2-3 posiłkach? TAK/NIE

TAK = 1 pkt

NIE = 0 pkt

SUMA = ………………………..

XI. ANALIZA KWESTIONARIUSZA FFQ-6

1. SŁODYCZE I PRZEKĄSKI

1. PROD. MLECZNE I JAJA

2. PROD. ZBOŻOWE

3. TŁUSZCZE

4. OWOCE

5. WARZYWA I ZIARNA

6. PROD. MIĘSNE I RYBY

7. NAPOJE

PODSUMOWANIE:
…………………………………………………………………………………
…………………………………………………………………………………
…………………………………………………………………………………

Uwagi:
…………………………………………………………………………………………
…………………………………………………………………………………………
…………………………………………………………………………………………
…………………………………………………………………………………………

QUESTIONNAIRE – FIRST VISIT "New Transplant"

Study ID Number: ____________________

I. GENERAL INFORMATION

Date: __________________ Study ID: __________________

Dietitian's initials: ____________ Patient's initials: ____________

Date of the next visit: __________________

II. PERSONAL, DEMOGRAPHIC AND SOCIOECONOMIC DATA

Age: __________________

Contact telephone number: __________________

Sex: ☐ Female ☐ Male

Place of residence:

City/Town: __________________

☐ Rural area

☐ Small town (<20,000 inhabitants)

☐ Medium-sized town (20,000–100,000 inhabitants)

☐ Large city (>100,000 inhabitants)

Education: ☐ Primary ☐ Secondary ☐ Vocational ☐ Higher

Monthly net income per family member: ☐ <1,000 PLN ☐ 1,000–2,500 PLN ☐ 2,501–4,000 PLN ☐ >4,000 PLN

Religion: ☐ Roman Catholic ☐ Orthodox ☐ Other ☐ No religious affiliation

III. TRANSPLANT-RELATED INFORMATION

Date of kidney transplantation: __________________

Type of donor: ☐ Deceased donor ☐ Living donor ☐ Unknown

Primary cause leading to transplantation:

☐ Kidney failure ☐ Diabetes ☐ Hypertension ☐ Glomerulonephritis ☐ Genetic disorder ☐ Other: ____________

Did you undergo dialysis before transplantation? ☐ Yes ☐ No

If yes, for how long? __________________

Was the donor a family member? ☐ Yes ☐ No

IV. COMORBIDITIES

Do you have any coexisting medical conditions? ☐ Yes ☐ No

If yes, we complete the table below using the medical records.

No.	Disease	ICD Code

V. CURRENT MEDICATIONS

Medication (immunosuppressive regimen / other medications)	Dose

VI. ANTHROPOMETRIC MEASUREMENTS

Body weight (kg): ____________

Height (cm): ____________

Waist circumference (cm): ____________

Hip circumference (cm): ____________

Calf circumference (cm): Right _____ Left _____

Mid-upper arm circumference (cm): ____________

VII. DIETARY HABITS AND LIFESTYLE

Do you eat fast food (e.g. hamburgers, pizza, hot dogs)? ☐ Yes ☐ No

If yes, how many times per month? ____________

Do you eat meals outside the home (restaurants, cafés)? ☐ Yes ☐ No

If yes, how many times per month? ____________

Do you use a meal delivery (diet catering) service? ☐ Yes ☐ No

If yes, how many times per month? ____________

Do you smoke cigarettes? ☐ Yes ☐ No

If yes, how many cigarettes per day? ____________

Do you drink coffee? ☐ Yes ☐ No

If yes, how many cups per day? ____________

VIII. BIOCHEMICAL TEST RESULTS (from medical records)

Parameter	Result
Albumin	
Prealbumin	
Total cholesterol	
HDL cholesterol	
Non-HDL cholesterol	
LDL cholesterol	
Triglycerides	
Fasting plasma glucose	
Uric acid	
Urea	
Creatinine	
Estimated glomerular filtration rate (eGFR)	
Tacrolimus trough concentration	
Serum magnesium	
Serum potassium	
Serum sodium	
Total serum calcium	
Iron	
25-hydroxyvitamin D (Vitamin D3)	
C-reactive protein (CRP)	
Thyroid-stimulating hormone (TSH)	
Parathyroid hormone (PTH)	
Other	

IX. BIOELECTRICAL IMPEDANCE ANALYSIS (BIA)

Test number: ____________

Fat mass (FAT, kg): ____________

Fat mass (%): ____________

Muscle mass (kg): ____________

Total body water (TBW, %): ____________

Extracellular water (ECW, %): ____________

Intracellular water (ICW, %): ____________

ECW/ICW ratio: ____________

Protein mass (kg): ____________

Mineral mass (kg): ____________

Body cell mass (BCM, kg): ____________

Extracellular mass (ECM, kg): ____________

Target body fat (%): ____________

Target body weight (kg): ____________

Target body water (%): ____________

X. ASSESSMENT OF THE 3-DAY FOOD DIARY

Food diary: ☐ Will be sent by e-mail ☐ Will be brought to the next visit ☐ Not determined

1. Does the patient consume 4–5 meals per day? ☐ Yes ☐ No

2. Are intervals between meals no longer than 4 hours? ☐ Yes ☐ No

3. Is the patient's diet varied? ☐ Yes ☐ No

4. Are fruit and vegetables consumed daily in 2–3 meals? ☐ Yes ☐ No

5. Are milk and dairy products consumed daily in 2–3 meals? ☐ Yes ☐ No

Yes = 1 point No = 0 points Total score: ____________

XI. FFQ-6 QUESTIONNAIRE ANALYSIS

- Sweets and snacks
- Milk and dairy products and eggs
- Cereal products
- Fats
- Fruit
- Vegetables and seeds

- Meat products and fish
- Beverages
- Summary
- Comments

Summary:

……………………………………………………………………………

……………………………………………………………………………

…………………………………………………………………………….

Comments:

……………………………………………………………………………

……………………………………………………………………………

…………………………………………………………………………….

……………………

Numer ID

„STARY PRZESZCZEP"

KWESTIONARIUSZ

I WIZYTA

I. INFORMACJE WSTĘPNE

**Data:**	**Numer ID:**
**Inicjały dietetyka:**	**Inicjały pacjenta:**

Data kolejnej wizyty: ……………………….

II. DANE OSOBOWE, DEMOGRAFICZNE I EKONOMICZNE

Wiek:	
Tel. kontaktowy:	

PŁEĆ

☐ KOBIETA	☐ MĘŻCZYZNA

MIEJSCE ZAMIESZKANIA

Nazwa miasta: …………………………

☐ WIEŚ	☐ MIASTO MAŁE (poniżej 20 tys. mieszkańców)	☐ MIASTO ŚREDNIE (20-100 tys. mieszkańców)	☐ MIASTO DUŻE (100 tys. mieszkańców)

WYKSZTAŁCENIE

☐ PODSTAWOWE	☐ ŚREDNIE	☐ ZAWODOWE	☐ WYŻSZE

MIESIĘCZNY DOCHÓD NA CZŁONKA RODZINY (netto)

☐ pon. 1000 zł	☐ 1000-2500 zł	☐ 2501-4000 zł	☐ pow. 4000 zł

WYZNANIE

☐ KOŚCIÓŁ KATOLICKI	☐ KOŚCIÓŁ PRAWOSŁAWNY	☐ INNE	☐ NIE NALEŻĘ DO ŻADNEGO WYZNANIA

III. INFORMACJE DOTYCZĄCE PRZESZCZEPU

DATA PRZESZCZEPU:	

CZY PRZESZCZEP BYŁ OD ZMARŁEGO CZY OD ŻYWEGO DAWCY?

☐ ZMARŁY DAWCA	☐ ŻYWY DAWCA	☐ NIE WIADOMO

JAKA BYŁA PRZYCZYNA PRZESZCZEPU?

☐ NIEWYDOLNOŚĆ NEREK	☐ CUKRZYCA	☐ NADCIŚNIENIE
☐ ZAPALENIE KŁĘBUSZKÓW NEREK	☐ GENETYCZNA	☐ INNE

CZY BYŁ PAN/ PANI DIALIZOWANY/ DIALIZOWANA PRZED PRZESZCZEPEM?

☐ TAK	☐ NIE

Jeśli tak, to ile czasu: ……………………………………………………………

CZY PRZESZCZEP BYŁ OD CZŁONKA RODZINY?

☐ TAK	☐ NIE

IV. WYSTĘPOWANIE CHORÓB WSPÓŁISTNIEJĄCYCH

CHOROBY WSPÓŁISTNIEJĄCE

Czy występują u Pani/Pana jakieś choroby?

☐ TAK*	☐ NIE

*Jeśli tak, to uzupełniamy poniżej z dokumentacji medycznej

Lp.	NAZWA CHOROBY	KOD ICD
1.		
2.		
3.		
4.		
5.		
6.		
7.		

V. PRZYJMOWANE LEKI

**LEKI IMMUNOSUPRESYJNE**			**INNE LEKI**
Nazwa leku	Substancja czynna	Dawkowanie	

VI. POMIARY ANTROPOMETRYCZNE

WAGA [kg]:	WZROST [cm]:
OBWÓD TALII [cm]:	OBWÓD BIODER [cm]:

OBWÓD ŁYDKI [cm]:	P:
	L:
OBWÓD RAMIENIA [cm]:	

VII. DIETA I UŻYWKI

Czy spożywa Pan/Pani fast-foody (typu hamburgery, pizza, hot-dogi)?

☐ TAK	☐ NIE

Jeśli tak, to jak często w miesiącu? ……………………………………………..

Czy zdarza się Panu/Pani jeść „na mieście" (typu restauracje, kawiarnie)?

☐ TAK	☐ NIE

Jeśli tak, to jak często w miesiącu? ………………………………………………..

Czy korzysta Pan/Pani z cateringu dietetycznego?

☐ TAK	☐ NIE

Jeśli tak, to jak często w miesiącu? ………………………………………………..

Czy pali Pan/Pani papierosy?

☐ TAK	☐ NIE

Jeśli tak, to ile [sztuk/dzień]? ..……………………………………………..

Czy spożywa Pan/ Pani kawę?

☐ TAK	☐ NIE

Jeśli tak, to ile [filiżanek/dzień]? ..……………………………………….

VIII. WYNIKI BADAŃ BIOCHEMICZNYCH

(*(z dokumentacji medycznej)*

Lp.	Nazwa
1.	ALBUMINY:
2.	PREALBUMINY:
3.	CHOLESTEROL CAŁKOWITY:
4.	CHOLESTEROL HDL:
5.	CHOLESTEROL NIE-HDL:
6.	CHOLESTEROL LDL:
7.	TRIGLICERYDY:
8.	GLUKOZA NA CZCZO:
9.	KWAS MOCZOWY:
10.	MOCZNIK:
11.	KREATYNINA
12.	eGFR:
13.	TACROLIMUS:
14.	Magnez (Mg) w surowicy:
15.	Potas (K) w surowicy:
16.	Sód (Na) w surowicy:
17.	Wapń całkowity w surowicy:
18.	Żelazo (Fe) :
19.	Witamina D_3:

20.	BIAŁKO CRP:
21.	TSH:
22.	PTH:
23.	INNE:

IX. BADANIE BIOIMPEDANCJI ELEKTRYCZNEJ (BIA)

Nr badania: ……

FAT [kg] =<br>FAT [%] =<br>Musc [kg] =<br>TBW [%] =<br>ECW [%] =<br>ICW [%] =<br>ECW/ICW =	Prot [kg] =<br>Mnrl [kg] =<br>BCM [kg] =<br>ECM [kg] =<br><br>TARGETS<br>• T. Fat % =<br>• T. Wgt kg =<br>• T. Wtr % =

X. OCENA 3-DNIOWEGO DZIENNICZKA SPOŻYCIA

☐ prześle drogą mailową	☐ przyniesie na kolejną wizytę	☐ nie ustalono

OCENA JAKOŚCIOWA DZIENNICZKA

1. Czy liczba posiłków wynosi 4-5 dziennie? TAK/NIE
2. Czy przerwy między posiłkami wynoszą nie więcej niż 4 h? TAK/NIE
3. Czy dieta pacjenta jest urozmaicona? TAK/NIE

4. Czy w diecie pacjenta owoce/ warzywa występują codziennie w 2-3 posiłkach? TAK/NIE
5. Czy w diecie pacjenta mleko i produkty mleczne występują codziennie w 2-3 posiłkach? TAK/NIE

TAK = 1 pkt

NIE = 0 pkt

SUMA = ………………………..

XI. ANALIZA KWESTIONARIUSZA FFQ-6

1. SŁODYCZE I PRZEKĄSKI

1. PROD. MLECZNE I JAJA

2. PROD. ZBOŻOWE

3. TŁUSZCZE

4. OWOCE

5. WARZYWA I ZIARNA

6. PROD. MIĘSNE I RYBY

7. NAPOJE

PODSUMOWANIE:
………………………………………………………………………………
………………………………………………………………………………
………………………………………………………………………………

Uwagi:
…………………………………………………………………………………………
…………………………………………………………………………………………
…………………………………………………………………………………………
…………………………………………………………………………………………

QUESTIONNAIRE – FIRST VISIT “Old Transplant”

Study ID Number: ____________________

I. GENERAL INFORMATION

Date: ____________ Study ID: ____________

Dietitian's initials: ____________ Patient's initials: ____________

Date of the next visit: ____________________

II. PERSONAL, DEMOGRAPHIC AND SOCIOECONOMIC DATA

Age: __________________

Contact telephone number: __________________

Sex: ☐ Female ☐ Male

Place of residence: City/Town __________________

☐ Rural area ☐ Small town (<20,000 inhabitants) ☐ Medium-sized town (20,000–100,000 inhabitants) ☐ Large city (>100,000 inhabitants)

Education: ☐ Primary ☐ Secondary ☐ Vocational ☐ Higher

Monthly net income per family member: ☐ <1,000 PLN ☐ 1,000–2,500 PLN ☐ 2,501–4,000 PLN ☐ >4,000 PLN

Religion: ☐ Roman Catholic ☐ Orthodox ☐ Other ☐ No religious affiliation

III. TRANSPLANT-RELATED INFORMATION

Date of kidney transplantation: __________________

Donor type: ☐ Deceased donor ☐ Living donor ☐ Unknown

Primary cause leading to transplantation: ☐ Kidney failure ☐ Diabetes ☐ Hypertension ☐ Glomerulonephritis ☐ Genetic disorder ☐ Other

Did you undergo dialysis before transplantation? ☐ Yes ☐ No

If yes, duration of dialysis: __________________

Was the donor a family member? ☐ Yes ☐ No

IV. COMORBIDITIES

Do you have any comorbidities? ☐ Yes ☐ No

If yes, complete the table below using the medical records.

No.	Disease	ICD Code

V. CURRENT MEDICATIONS

Immunosuppressive medication	Active substance	Dose

VI. ANTHROPOMETRIC MEASUREMENTS

Body weight (kg): ____________

Height (cm): ____________

Waist circumference (cm): ____________

Hip circumference (cm): ____________

Calf circumference (cm): Right ____ Left ____: ____________

Mid-upper arm circumference (cm): ____________

VII. DIETARY HABITS AND LIFESTYLE

Do you eat fast food (e.g. hamburgers, pizza, hot dogs)? ☐ Yes ☐ No

If yes, how many times per month? ____________

Do you eat out (restaurants, cafés)? ☐ Yes ☐ No

If yes, how many times per month? ____________

Do you use a meal delivery (diet catering) service? ☐ Yes ☐ No

If yes, how many times per month? ____________

Do you smoke cigarettes? ☐ Yes ☐ No

If yes, cigarettes per day: ____________

Do you drink coffee? ☐ Yes ☐ No

If yes, cups per day: ____________

VIII. BIOCHEMICAL TEST RESULTS (from medical records)

Parameter	Result
Albumin	
Prealbumin	
Total cholesterol	
HDL cholesterol	
Non-HDL cholesterol	
LDL cholesterol	
Triglycerides	
Fasting plasma glucose	
Uric acid	
Urea	
Creatinine	
Estimated glomerular filtration rate (eGFR)	
Tacrolimus trough concentration	
Serum magnesium	
Serum potassium	
Serum sodium	
Total serum calcium	
Iron	
25-hydroxyvitamin D (Vitamin D3)	
C-reactive protein (CRP)	
Thyroid-stimulating hormone (TSH)	
Parathyroid hormone (PTH)	
Other	

IX. BIOELECTRICAL IMPEDANCE ANALYSIS (BIA)

Test number: ____________

Fat mass (FAT, kg): ____________

Fat mass (%): ____________

Muscle mass (kg): ____________

Total body water (TBW, %): ____________

Extracellular water (ECW, %): ____________

Intracellular water (ICW, %): ____________

ECW/ICW ratio: ____________

Protein mass (kg): ____________

Mineral mass (kg): ____________

Body cell mass (BCM, kg): ____________

Extracellular mass (ECM, kg): ____________

Target body fat (%): ____________

Target body weight (kg): ____________

Target body water (%): ____________

X. ASSESSMENT OF THE 3-DAY FOOD DIARY

Food diary: ☐ Will be sent by e-mail ☐ Will be brought to the next visit ☐ Not determined

1. Does the patient consume 4–5 meals per day? ☐ Yes ☐ No

2. Are intervals between meals no longer than 4 hours? ☐ Yes ☐ No

3. Is the patient's diet varied? ☐ Yes ☐ No

4. Are fruit and vegetables consumed daily in 2–3 meals? ☐ Yes ☐ No

5. Are milk and dairy products consumed daily in 2–3 meals? ☐ Yes ☐ No

Yes = 1 point No = 0 points Total score: ____________

XI. FFQ-6 QUESTIONNAIRE ANALYSIS

• Sweets and snacks

• Milk and dairy products and eggs

• Cereal products

• Fats

• Fruit

• Vegetables and seeds

• Meat products and fish

• Beverages

• Summary

• Comments

Summary:

…………………………………………………………………………………

…………………………………………………………………………………

…………………………………………………………………………………..

Comments:

…………………………………………………………………………………

…………………………………………………………………………………

…………………………………………………………………………………..

Supplementary File S3

OCENA RYZYKA ZWIĄZANEGO ZE STANEM ODŻYWIENIA (NUTRITIONAL RISK SCORE, NRS 2002)

**Pogorszenie stanu odżywienia**		**Nasilenie choroby (zwiększone zapotrzebowanie)**	
Brak = 0	Prawidłowy stan odżywienia	Brak = 0	Zwykłe zapotrzebowanie
Lekkie = 1	Utrata masy ciała > 5 % w ciągu 3 miesięcy lub<br>Spożycie pokarmu 50-75% w ostatnim tygodniu	Lekkie = 1	Np. złamanie uda<br>Choroby przewlekłe (zwłaszcza powikłane) - marskość wątroby, POChP<br>Radioterapia
Średnie = 2	Utrata masy ciała > 5% w ciągu 2 miesięcy lub<br>BMI 18,5-20,5 + zły stan ogólny lub<br>Spożycie pokarmu 25-50% potrzeb w ostatnim tygodniu	Średnie = 2	Rozległe operacje brzuszne<br>Udar mózgu<br>Chorzy w wieku podeszłym – leczenie przewlekłe<br>Pooperacyjna niewydolność nerek<br>Chemioterapia
Ciężkie = 3	Utrata masy ciała > 5 % w ciągu 1 miesiąca lub<br>BMI <18,5 + zły stan ogólny lub<br>Spożycie pokarmu 0-25% potrzeb w ostatnim tygodniu	Ciężkie = 3	Uraz głowy<br>Przeszczep szpiku<br>Chory w oddziale Intensywnej Terapii
**Jeżeli wiek chorego przekracza 70 lat + 1 pkt**			
Nasilenie:		Nasilenie:	Suma punktów:
Wynik: ≥3 – wskazane leczenie żywieniowe<br><3 – rozważ postępowanie zachowawcze, powtórz badanie za tydzień.			

……………………………………… ……………………………………………

podpis i pieczęć lekarza leczącego podpis i pieczęć członka zespołu żywienia

NUTRITIONAL RISK SCREENING (NRS-2002)

Impaired Nutritional Status		Disease Severity (Increased Nutritional Requirements)	
None = 0	Normal nutritional status	None = 0	Normal nutritional requirements
Mild = 1	Weight loss >5% within 3 months OR food intake 50–75% of normal requirement during the previous week	Mild = 1	Hip fracture; chronic diseases (especially with complications), liver cirrhosis, COPD; radiotherapy
Moderate = 2	Weight loss >5% within 2 months OR BMI 18.5–20.5 kg/m² with impaired general condition OR food intake 25-50% during the previous week	Moderate = 2	Major abdominal surgery; stroke; elderly patients with chronic diseases; postoperative renal failure; chemotherapy
Severe = 3	Weight loss >5% within 1 month OR BMI <18.5 kg/m² with impaired general condition OR food intake 0–25% during the previous week	Severe = 3	Head injury; bone marrow transplantation; Intensive Care Unit patient
If the patient is ≥70 years old: add 1 point			
Nutritional status:	Disease severity:	Total score:	
Result: ≥3 – nutritional support is indicated.<br><3 – consider conservative management and repeat screening after 1 week.			

Signature and stamp of attending physician

Signature and stamp of nutrition team member

Supplementary File S4

Kwestionariusz częstotliwości spożycia żywności FFQ-6

Prowadzimy badania na temat odżywiania i zdrowia.
Proszę odpowiedzieć na wszystkie pytania najlepiej jak Pan(i) potrafi.
Jeśli nie umie Pan(i) odpowiedzieć, to proszę określić w przybliżeniu.
Taka odpowiedź będzie dla nas bardziej pomocna niż brak odpowiedzi.
Udzielone informacje są poufne i będą wykorzystane jedynie w celach naukowych.

Komentarz i instrukcje dla ankietera
Ankieter odczytuje pytanie, możliwe odpowiedzi i zapisuje odpowiedź wybraną przez Respondenta We wskazanych pytaniach ankieter pokazuje Respondentowi karty odpowiedzi

1. Numer kodowy (wypełnia ankieter) ☐☐☐☐

2. Płeć 1. Mężczyzna ☐ 2. Kobieta ☐

3. Data badania (dd/mm/rrrr) ☐☐ ☐☐ ☐☐☐☐

4. Data urodzenia (dd/mm/rrrr) ☐☐ ☐☐ ☐☐☐☐

Proszę powiedzieć jak często jadł(a) Pan(i) następujące produkty?
Proszę wybrać ***jedną*** *odpowiedź najlepiej opisującą Pana(i) sposób odżywiania w ciągu ostatnich 12 miesięcy.*
Proszę wziąć pod uwagę wszystkie posiłki i pojadanie, jedzone w domu i poza nim.

Pokazać kartę odpowiedzi A

**Jak często w ciągu ostatnich 12 miesięcy pił(a) lub jadł(a) Pan(i):**	Nigdy lub prawie nigdy	Raz w miesiącu lub rzadziej	Kilka razy w miesiącu	Kilka razy w tygodniu	Codziennie	Kilka razy dziennie
Kody częstości spożycia:	*(1)*	*(2)*	*(3)*	*(4)*	*(5)*	*(6)*
**Słodycze i przekąski**						bbbb
**5. Cukier** do słodzenia napojów						
**6. Miód** do potraw i słodzenia napojów						
**7. Czekolady, cukierki czekoladowe i batony czekoladowe**						
**8. Cukierki nie czekoladowe**, np. cukierki owocowe, karmelki, żelki, krówki, toffi						
**9. Herbatniki i ciastka**, np. kruche, półkruche, ciastka z kremem, z owocami, drożdżowe, serniki, pączki, makowiec, drożdżówki, mufinki, croissanty						
**10. Lody i budyń**						

Oprac. prof. dr hab. inż. Lidia Wądołowska i dr inż. Ewa Niedźwiedzka www.uwm.edu.pl/edu/lidiawadolowska

**11. Przekąski słone,** np. chipsy, solone chrupki, krakersy, paluszki						

**Jak często w ciągu ostatnich 12 miesięcy pił(a) lub jadł(a) Pan(i):**	Nigdy lub prawie nigdy	Raz w miesiącu lub rzadziej	Kilka razy w miesiącu	Kilka razy w tygodniu	Codziennie	Kilka razy dziennie
Kody częstości spożycia:	*(1)*	*(2)*	*(3)*	*(4)*	*(5)*	*(6)*
**Produkty mleczne i jaja**						
**12. Mleko i naturalne napoje mleczne**, np. mleko, zupy mleczne, jogurt naturalny, kefir, maślanka naturalna						
**13. Napoje mleczne słodzone**, np. jogurty owocowe, jogurty z płatkami czekoladowymi, maślanka smakowa, owocowy jogurt, kakao na mleku						
**14. Twarogi naturalne**, np. sery twarogowe różne, twarożki naturalne, mozarella, serki twarogowe z ziołami						
**15. Twarożki smakowe**, np. owocowe, czekoladowe, waniliowe						
**16. Sery**, np. sery żółte, pleśniowe, sery topione, serki do smarowania						
**17. Jaja i potrawy z jaj, w których jaja stanowią podstawowy składnik potrawy,** np. jajecznica, omlet, pasta z jaj, jaja gotowane						
**Produkty zbożowe**						
**18. Pieczywo razowe** lub **z ziarnami, tzw. ciemne** np. chleb razowy żytni, graham, chleb pszenny lub żytni z ziarnami, pumpernikiel, grahamki, pieczywo chrupkie						
**19. Pieczywo rafinowane, tzw. jasne,** np. chleb jasny pszenny lub żytni, pszenno-żytni, pieczywo tostowe, bułki zwykłe, bułki i rogale maślane						
**20. Kasze gruboziarniste nie rafinowane**, np. kasza gryczana, pęczak, **ryż brązowy, makaron razowy**						
**21. Kasze drobnoziarniste rafinowane**, np. manna, jęczmienna łamana, **makaron, ryż biały**, płatki ryżowe						
**22. Gotowe śniadaniowe produkty zbożowe, tj. nie gotowane dodatki do mleka, np. muesli, płatki kukurydziane, z dodatkami lub bez**						

**Jak często w ciągu ostatnich 12 miesięcy pił(a) lub jadł(a) Pan(i):**	Nigdy lub prawie nigdy	Raz w miesiącu lub rzadziej	Kilka razy w miesiącu	Kilka razy w tygodniu	Codziennie	Kilka razy dziennie
Kody częstości spożycia:	*(1)*	*(2)*	*(3)*	*(4)*	*(5)*	*(6)*
**Tłuszcze**						
**23. Olej,** wszystkie rodzaje						
**24. Masło,** wszystkie rodzaje						
**25. Margaryna w kostkach** (do pieczenia, smażenia), **margaryna w kubeczkach** (do smarowania), wszystkie rodzaje						
**26. Śmietana, śmietanka,** ukwaszona lub słodka, do potraw lub napojów						
**27. Inne tłuszcze zwierzęce**, np. smalec, słonina						
**28. Majonez i dresingi**, tj. sosy sałatkowe – wszystkie rodzaje						
**Owoce**						
**29. Owoce, wszystkie rodzaje** Zapytać oddzielnie o owoce ogółem, a potem o różne rodzaje owoców; „pytanie weryfikujące”						
**30. Owoce pestkowe**, np. morele, avocado, wiśnie, czereśnie, nektarynki, brzoskwinie, śliwki, winogrona						
**31. Kiwi i cytrusy**, np. pomarańcze, mandarynki, grejpfruty, cytryny, pomelo						
**32. Owoce tropikalne pozostałe**, np. ananas, arbuz, melon, daktyle świeże, figi świeże						
**33. Owoce jagodowe**, np. truskawki, maliny, jeżyny, czarna jagoda, porzeczki						
**34. Banany**						
**35. Jabłka i gruszki**						
**36. Awokado**						
**37. Oliwki**						
**38. Owoce suszone**, np. rodzynki, morele, figi, jabłka, śliwki						
**39. Słodkie przetwory owocowe** i **owoce kandyzowane**, np. kompoty owocowe, dżemy, powidła, konfitury, daktyle, suszone banany						

**Jak często w ciągu ostatnich 12 miesięcy pił(a) lub jadł(a) Pan(i):**	Nigdy lub prawie nigdy	Raz w miesiącu lub rzadziej	Kilka razy w miesiącu	Kilka razy w tygodniu	Codziennie	Kilka razy dziennie
Kody częstości spożycia:	*(1)*	*(2)*	*(3)*	*(4)*	*(5)*	*(6)*
**Warzywa i ziarna**						
**40. Warzywa, wszystkie rodzaje** Zapytać oddzielnie o warzywa ogółem, a potem o różne rodzaje warzyw; „pytanie weryfikujące”						
**41. Warzywa krzyżowe**, np. kapusta biała, włoska, czerwona, pekińska, chińska, brukselka, kalafior, brokuły, jarmuż						
**42. Warzywa żółto-pomarańczowe**, np. marchew, papryka						
**43. Warzywa zielone liściaste**, np. szpinak, cykoria, sałata masłowa, sałata lodowa, sałata rzymska, rukola, pory, seler naciowy, liście pietruszki						
**44. Pomidory**						
**45. Warzywa takie jak ogórek**, ogórki świeże, kabaczek, cukinia, dynia, bakłażan						
**46. Warzywa korzeniowe i pozostałe**, np. pietruszka korzeń, buraki czerwone, pasternak, cebula, czosnek, seler korzeniowy, rzodkiew, rzepa, sałatki i surówki z warzyw mieszanych						
**47. Świeże nasiona roślin strączkowych i w puszce**, np. kukurydza, groszek zielony, fasolka szparagowa, fasolka zielona						
**48. Suche nasiona roślin strączkowych** np. bób, fasola, soja, groch, ciecierzyca, **i w potrawach**, np. grochówka, fasolka po bretońsku, humus i inne pasty						
**49. Ziemniaki,** w różnej postaci, np. gotowane, pieczone, frytki, placki ziemniaczane, kopytka						
**50. Orzechy**, np. ziemne, laskowe, włoskie, migdały, pistacje, nerkowca, orzech kokosowy, kasztan, **kremy orzechowe**, np. masło orzechowe, krem czekoladowo-orzechowy,						
**51. Ziarna, np. dyni, sezamu, słonecznika, zarodki pszenne**						

**Jak często w ciągu ostatnich 12 miesięcy pił(a) lub jadł(a) Pan(i):**	Nigdy lub prawie nigdy	Raz w miesiącu lub rzadziej	Kilka razy w miesiącu	Kilka razy w tygodniu	Codziennie	Kilka razy dziennie
Kody częstości spożycia:	*(1)*	*(2)*	*(3)*	*(4)*	*(5)*	*(6)*
**Produkty mięsne i ryby**						
**52. Kiełbasy**, różne rodzaje, np. mielonka, parówki, toruńska, zwyczajna, wiejska, żywiecka, krakowska sucha, boczek, bekon						
**53. Wędliny wysokogatunkowe**, np. szynka, polędwica – drobiowe, wieprzowe, wołowe i mieszane						
**54. Wyroby wędliniarskie i mięso narządowe**, np. wątróbka, kaszanka, móżdżek, salceson, pasztety						
**55. Mięso czerwone**, np. mięso wieprzowe, wołowe, cielęce						
**56. Mięso drobiowe i z królika**, np. mięso drobiowe z kury, kurczaka, kaczki, indyka						
**57. Dziczyzna**, np. mięso z dzika, sarny, przepiórki, dzikiej kaczki, zająca						
**58. Ryby chude**, np. mintaj, dorsz, okoń, morszczuk, karp do 1 kg, tuńczyk, panga, pstrąg						
**59. Ryby tłuste**, np. łosoś, sardynki, śledź, makrela, karp duży, węgorz						
**Napoje**						
**60. Soki owocowe i nektary owocowe,** np. wieloowocowe, pomarańczowy, grejpfrutowy, jabłkowy, gruszkowy, winogronowy, z czarnej porzeczki, aronii, wiśniowy						
**61. Soki warzywne i warzywno-owocowe,** np. z warzyw mieszanych, pomidorowy, marchwiowy, marchwiowo-owocowy						
**62. Napoje energetyzujące** Pokazać kartę odpowiedzi B						
**63. Słodzone napoje** typu Fanta, Coca-cola, Mirinda, Sprite itp.						
**64. Piwo**						
**65. Wino i drinki**						
**66. Wódkę i alkohole wysokoprocentowe**						

Dziękujemy za udzielenie odpowiedzi i poświęcony czas

Oprac. prof. dr hab. inż. Lidia Wądołowska i dr inż. Ewa Niedźwiedzka www.uwm.edu.pl/edu/lidiawadolowska

Terminologia i opis kwestionariusza częstotliwości spożycia żywności FFQ-6

Kwestionariusz częstotliwości spożycia żywności o akronimie FFQ-6 (*Food Frequency Questionnaire*) służy do zebrania informacji o częstotliwości spożywania 62 asortymentowych grup produktów, reprezentujących 8 głównych grup żywności:

1. Słodycze i przekąski:
- Cukier
- Miód
- Czekolady, cukierki czekoladowe i batony czekoladowe
- Cukierki nie czekoladowe
- Herbatniki i ciastka
- Lody i budyń
- Przekąski słone

2. Produkty mleczne i jaja:
- Mleko i naturalne napoje mleczne
- Napoje mleczne słodzone
- Twarogi naturalne
- Twarożki smakowe
- Sery
- Jaja i potrawy z jaj

3. Produkty zbożowe:
- Pieczywo razowe lub z ziarnami
- Pieczywo rafinowane
- Kasze gruboziarniste nie rafinowane
- Kasze drobnoziarniste rafinowane
- Gotowe śniadaniowe produkty zbożowe

4. Tłuszcze:
- Olej
- Masło
- Margaryna w kostkach i kubeczkach
- Śmietana śmietanka
- Inne tłuszcze zwierzęce
- Majonez i dresingi

5. Owoce:
- Owoce, wszystkie rodzaje
- Owoce pestkowe
- Kiwi i cytrusy
- Owoce tropikalne pozostałe
- Owoce jagodowe
- Banany
- Jabłka i gruszki
- Awokado
- Oliwki
- Owoce suszone
- Słodkie przetwory owocowe i owoce kandyzowane

6. Warzywa i ziarna:
- Warzywa, wszystkie rodzaje
- Warzywa krzyżowe
- Warzywa żółto-pomarańczowe
- Warzywa zielone liściaste
- Pomidory
- Warzywa takie jak ogórek
- Warzywa korzeniowe i pozostałe
- Świeże nasiona roślin strączkowych i w puszce
- Suche nasiona roślin strączkowych i w potrawach
- Ziemniaki
- Orzechy i kremy orzechowe
- Ziarna
- Pozostałe warzywa

7. Produkty mięsne i ryby:
- Kiełbasy
- Wędliny wysokogatunkowe
- Wyroby wędliniarskie i mięso narządowe
- Mięso czerwone
- Mięso drobiowe i z królika
- Dziczyzna
- Ryby chude
- Ryby tłuste

8. Napoje:
- Soki owocowe i nektary owocowe
- Soki warzywne i warzywno-owocowe
- Napoje energetyzujące
- Napoje słodzone
- Piwo
- Wino i drinki
- Wódka i alkohole wysokoprocentowe

Kwestionariusz FFQ-6 składa się z dwóch części. Pierwsza część zwiera tzw. metryczkę, czyli pytania o płeć, datę urodzenia respondenta i datę badania.

Zasadnicza część kwestionariusza FFQ-6 dotyczy częstotliwości spożycia 62 asortymentowych grup produktów żywnościowych spożywanych w ciągu ostatnich 12 miesięcy. Listę produktów i ich podział na grupy żywności opracowano korzystając z doświadczeń własnych i wzorowano na kalibrowanym kwestionariuszu urozmaicenia spożycia żywności (FIVeQ) (Niedźwiedzka, Wądołowska 2008; Niedźwiedzka 2009). Kwestionariusz FFQ-6 zawiera dwa pytania dotyczące częstotliwości spożycia warzyw ogółem i owoców ogółem, które ułatwiają weryfikację i interpretację informacji o spożyciu asortymentowych grup warzyw i owoców, ponieważ wielu respondentów ma tendencję do przeszacowania spożycia, jeśli kwestionariusz zawiera wiele szczegółowych pytań dotyczących żywności o ogólnie znanych cechach prozdrowotnych. Respondenci mają do wyboru 6 kategorii częstotliwości spożycia żywności: (1) nigdy lub prawie nigdy, (2) raz w miesiącu lub rzadziej, (3) kilka razy w miesiącu, (4) kilka razy w tygodniu, (5) codziennie, (6) kilka razy dziennie.

Interpretacja wyników kwestionariusza FFQ-6

Uzyskane informacje mają format danych jakościowych i mogą być opracowywane, a następnie interpretowane w następujący sposób:

- przez zastosowanie analizy rozkładów dla oryginalnych sześciu kategorii częstotliwości spożycia żywności,
- przez przekształcenie oryginalnych kategorii częstotliwości spożycia żywności w inne kategorie (łączenie kategorii),
- przez przekształcenie oryginalnych kategorii częstotliwości spożycia żywności w dane „półilościowe”, które logicznie odzwierciedlają rosnące natężenie cechy (tabela) stosując:
  - ich rangowanie, tj. przypisanie kategoriom częstotliwości spożycia żywności umownych liczb całkowitych,
  - przekształcenie w liczby o charakterze liczb rzeczywistych i wyrażenie częstotliwości spożycia żywności jako krotność/dzień.

Tabela. Wskaźniki zastosowane dla kategorii częstotliwości spożycia żywności kwestionariusza FFQ-6

Kategorie częstotliwości spożycia	Rangi przypisane kategoriom częstotliwości	Częstotliwość dzienna (krotność/dzień)
Nigdy lub prawie nigdy	1	0,0
Raz w miesiącu lub rzadziej	2	0,025
Kilka razy w miesiącu	3	0,100
Kilka razy w tygodniu	4	0,571
Codziennie	5	1,000
Kilka razy dziennie	6	2,000

Opis wyników walidacji kwestionariusza FFQ-6

Kwestionariusz FFQ-6 zwalidowano, a wyniki tego postępowania opracowano i aktualnie są przygotowywane do opublikowania (stan na 2.03.2014).

Ocenę niezawodności kwestionariusza FFQ-6 przeprowadzono w grupie młodych dziewcząt w wieku od 13 do 21 lat. Niezawodność kwestionariusza zmierzono poprzez porównanie wyników pierwszego wywiadu (test) i wywiadu powtórzonego z respondentem przez tego samego ankietera po okresie dwóch tygodni (retest). W ocenie niezawodności pytań kwestionariusza FFQ-6 (porównanie test-retest) zastosowano współczynnik korelacji Spearmana i statystykę kappa. Wysokie wartości współczynnika korelacji Spearmana (od 0,5 do 0,7) stwierdzono dla 26 grup żywności (42% ogólnej liczby produktów), zaś bardzo wysokie wartości współczynnika korelacji (od 0,7 do 0,9) uzyskano dla 31 grup żywności (50% ogólnej liczby produktów). Wartości statystyki kappa wynosiły od 0,32 do 0,72. Dobrą zgodność testu i retestu (wartości statystyki kappa od 0,6 do 0,8) stwierdzono dla 10 grup żywności (16% ogólnej liczby produktów), zaś umiarkowaną zgodność (kappa od 0,4 do 0,6) dla 50 grup żywności (81% ogólnej liczby produktów). Małą zgodność testu i retestu (kappa poniżej 0,4) uzyskano m.in. dla warzyw korzeniowych i pozostałych, lodów i budyniu, gotowych śniadaniowych produktów zbożowych, twarogów naturalnych, suchych nasion roślin strączkowych i w potrawach, wyrobów wędliniarskich i mięsa narządowego, a najwyższe wartości statystyki kappa uzyskano dla wódki i alkoholi wysokoprocentowych, wina i drinków, oliwek, piwa, tłuszczów zwierzęcych innych, napojów energetyzujących, pomidorów, owoców ogółem, warzyw zielonych liściastych, czekolady, cukierków czekoladowych i batonów czekoladowych.

Sposób cytowania źródła kwestionariusza FFQ-6:

- http://www.uwm.edu.pl/edu/lidiawadolowska/

Trwają prace nad opublikowaniem wyników walidacji FFQ-6 (Niedźwiedzka E. i in., 2014). Do czasu opublikowania wyników walidacji kwestionariusz FFQ-6 należy cytować podając stronę dostępu, a w metodyce własnej pracy należy podać wyniki walidacji zamieszczone powyżej (Opis wyników walidacji kwestionariusza FFQ-6).

Piśmiennictwo:

Bland J.M., Altman D.G.: Agreement between methods of measurement with multiple observations per individual. J. Biopharm. Stat., 2007, 17, 571-582.

Cade J., Thompson R., Burley V., Warm D.: Development, validation and utilisation of food-frequency questionnaires – a review. Pub. Health Nutr., 2002, 5, 567-587.

Elmadfa I., Freisling H.: Fat intake, diet variety and health promotion. Forum Nutr., 2005, 57, 1-10.

Horwath C., Kouris-Blazos A., Savige G., Wahlqvist M.L.: Eating your way to a successful old age, with special reference to older women. Asia Pacific J. Clin. Nutr., 1999, 8 (3), 216-225.

Jędrychowski W.: Metoda zbierania wywiadów lekarskich i budowa kwestionariuszy zdrowotnych. PZWL, Warszawa, 1982.

Niedźwiedzka E., Wądołowska L.: Accuracy analysis of the Food Intake Variety Questionnaire (FIVeQ). Reproducibility assessment among older people. Pakistan J. Nutr., 2008, 7, 3, 426-435.

Niedźwiedzka E.: Analiza urozmaicenia spożycia żywności i jego powiązań ze stanem odżywienia i ryzykiem niedożywienia u osób starszych. Rozprawa doktorska, Katedra Żywienia Człowieka, UWM Olsztyn, 2009 (maszynopis).

Niedźwiedzka E., Wądołowska L., Słowińska A., Kwakowicz M.: Kalibracja kwestionariusza urozmaicenia spożycia żywności (FIVeQ) w odniesieniu do metody 7-dniowego bieżącego notowania. Mat. VIII Krajowych Warsztatów Żywieniowych pt. „Metody ankietowe w badaniach żywieniowych", Mrózek k/Olsztynka, 2008, s. 55.

Niedźwiedzka E., Wądołowska L.: Analiza urozmaicenia spożycia żywności i jego powiązań ze stanem odżywienia polskich seniorów. Mat. VI Seminarium Środowiskowego Młodych Pracowników Nauki pt. „Bezpieczeństwo i jakość żywności", Olsztyn, Wyd. UWM, 2009, s. 20-21.

Wądołowska L.: Walidacja metod i mierniki statystyczne w badaniach sposobu żywienia. Rozdz. 3 (w:) Przewodnik metodyczny badań sposobu żywienia. Red. Gronowska-Senger A., Wyd. Komitetu Nauki o Żywieniu Człowieka Polskiej Akademii Nauk, Warszawa, 2013, 17-37.

Wądołowska L.: Zasady obliczania i interpretacji wyników. Rozdz. 4 (w:) Przewodnik metodyczny badań sposobu żywienia. Red. Gronowska-Senger A., Wyd. Komitetu Nauki o Żywieniu Człowieka Polskiej Akademii Nauk, Warszawa, 2013, 38-67. http://www.knozc.pan.pl/index.php/37-uncategorised/89-przewodnik-metodyczny-badan-sposobu-zywienia

Food Frequency Questionnaire (FFQ-6)

Developed by Prof. Lidia Wądołowska, PhD, DSc, Eng. and Ewa Niedźwiedzka, PhD, Eng.

We are conducting a study on nutrition and health.
Please answer all questions as accurately as you can.
If you are unsure of an answer, please provide your best estimate.
An approximate answer is more helpful than leaving a question unanswered.
All information provided will remain confidential and will be used solely for scientific research purposes.

Comments and Instructions for the Interviewer

The interviewer should read each question together with all possible response options and record the participant's selected answer. For designated questions, the interviewer should show the respondent the appropriate response cards.

1. Study ID (completed by the interviewer): ____________________

2. Sex: ☐ Male ☐ Female

3. Date of examination (dd/mm/yyyy): ____________________

4. Date of birth (dd/mm/yyyy): ____________________

Show Response Card A

During the past 12 months, how often did you consume the following foods and beverages?

Food item	Never (1)	Once/month or less (2)	Several /month (3)	Several /week (4)	Daily (5)	Several /day (6)
**Sweets and snacks**						
5. Sugar added to beverages	☐	☐	☐	☐	☐	☐
6. Honey used in foods or beverages	☐	☐	☐	☐	☐	☐
7. Chocolate, chocolate candies and chocolate bars	☐	☐	☐	☐	☐	☐
8. Non-chocolate confectionery	☐	☐	☐	☐	☐	☐
9. Biscuits, cookies and cakes	☐	☐	☐	☐	☐	☐
10. Ice cream and pudding	☐	☐	☐	☐	☐	☐

11. Salty snacks	☐	☐	☐	☐	☐	☐
**Dairy products and eggs**						
Food item	Never (1)	Once/month or less (2)	Several times/month (3)	Several times/week (4)	Daily (5)	Several times/day (6)
12. Milk and natural dairy products (milk, plain yogurt, kefir, buttermilk)	☐	☐	☐	☐	☐	☐
13. Sweetened dairy beverages (fruit yogurt, flavored buttermilk, cocoa with milk)	☐	☐	☐	☐	☐	☐
14. Plain curd cheese and fresh cheeses (including mozzarella, herb cottage cheese)	☐	☐	☐	☐	☐	☐
15. Flavored cottage cheese (fruit, chocolate, vanilla)	☐	☐	☐	☐	☐	☐
16. Cheese (hard, blue, processed, cream cheese spreads)	☐	☐	☐	☐	☐	☐
17. Eggs and egg-based dishes (scrambled eggs, omelet, egg spread, boiled eggs)	☐	☐	☐	☐	☐	☐
Cereal products						
18. Wholegrain or seeded bread (rye bread, graham bread, pumpernickel, crispbread)	☐	☐	☐	☐	☐	☐
19. Refined bread (white	☐	☐	☐	☐	☐	☐

bread, toast bread, wheat rolls, croissants)						
20. Wholegrain cereals and grains (buckwheat, pearl barley, brown rice, wholemeal pasta)	☐	☐	☐	☐	☐	☐
21. Refined cereals and grains (semolina, white rice, pasta, rice flakes)	☐	☐	☐	☐	☐	☐
22. Ready-to-eat breakfast cereals (muesli, cornflakes, breakfast cereals)	☐	☐	☐	☐	☐	☐
**Fats**						
23. Vegetable oils (all types)	☐	☐	☐	☐	☐	☐
24. Butter (all types)	☐	☐	☐	☐	☐	☐
25. Margarine (block or tub, all types)	☐	☐	☐	☐	☐	☐
26. Cream and sour cream	☐	☐	☐	☐	☐	☐
27. Other animal fats (e.g. lard)	☐	☐	☐	☐	☐	☐
28. Mayonnaise and salad dressings	☐	☐	☐	☐	☐	☐
**Fruits**						
29. Fruit (all types)	☐	☐	☐	☐	☐	☐
30. Stone fruits (e.g. apricots, cherries, peaches, plums, grapes, avocado)	☐	☐	☐	☐	☐	☐

31. Kiwi and citrus fruits (oranges, mandarins, grapefruit, lemons, pomelo)	☐	☐	☐	☐	☐	☐
32. Other tropical fruits (pineapple, watermelon, melon, fresh dates, fresh figs)	☐	☐	☐	☐	☐	☐
33. Berries (strawberries, raspberries, blackberries, blueberries, currants)	☐	☐	☐	☐	☐	☐
34. Bananas	☐	☐	☐	☐	☐	☐
35. Apples and pears	☐	☐	☐	☐	☐	☐
36. Avocados	☐	☐	☐	☐	☐	☐
37. Olives	☐	☐	☐	☐	☐	☐
38. Dried fruits (raisins, dried apricots, figs, apples, prunes)	☐	☐	☐	☐	☐	☐
39. Sweet fruit preserves and candied fruits (jams, preserves, compotes, candied fruit)	☐	☐	☐	☐	☐	☐
**Vegetables and Seeds**						
40. Vegetables (all types)	☐	☐	☐	☐	☐	☐
41. Cruciferous vegetables (cabbage, Brussels sprouts, cauliflower, broccoli, kale)	☐	☐	☐	☐	☐	☐
42. Yellow-orange vegetables (e.g. carrots, peppers)	☐	☐	☐	☐	☐	☐
43. Green leafy	☐	☐	☐	☐	☐	☐

vegetables (spinach, chicory, lettuce, arugula, celery leaves, parsley)						
44. Tomatoes	☐	☐	☐	☐	☐	☐
45. Cucumbers, zucchini, squash and eggplant	☐	☐	☐	☐	☐	☐
46. Root and other vegetables (beetroot, onion, garlic, celery root, radish, mixed salads)	☐	☐	☐	☐	☐	☐
47. Fresh and canned legumes (sweet corn, green peas, green beans)	☐	☐	☐	☐	☐	☐
48. Dried legumes and legume dishes (beans, peas, soybeans, chickpeas, lentils, hummus)	☐	☐	☐	☐	☐	☐
49. Potatoes (boiled, baked, fries, potato pancakes, dumplings)	☐	☐	☐	☐	☐	☐
50. Nuts and nut spreads (peanuts, walnuts, almonds, pistachios, peanut butter, chocolate-hazelnut spread)	☐	☐	☐	☐	☐	☐
51. Seeds (pumpkin, sesame, sunflower, wheat germ)	☐	☐	☐	☐	☐	☐

**Meat and Fish Products**						
52. Sausages and processed meat products	☐	☐	☐	☐	☐	☐
53. High-quality cold cuts (ham, loin, poultry, pork or beef cold cuts)	☐	☐	☐	☐	☐	☐
54. Offal and processed meat products (liver, black pudding, pâté, head cheese)	☐	☐	☐	☐	☐	☐
55. Red meat (pork, beef, veal)	☐	☐	☐	☐	☐	☐
56. Poultry and rabbit meat	☐	☐	☐	☐	☐	☐
57. Game meat (wild boar, venison, quail, wild duck, hare)	☐	☐	☐	☐	☐	☐
58. Lean fish (e.g. cod, pollock, perch, hake, trout)	☐	☐	☐	☐	☐	☐
59. Fatty fish (e.g. salmon, sardines, herring, mackerel, eel)	☐	☐	☐	☐	☐	☐
Beverages						
60. Fruit juices and fruit nectars	☐	☐	☐	☐	☐	☐
61. Vegetable and vegetable-fruit juices	☐	☐	☐	☐	☐	☐
62. Energy drinks	☐	☐	☐	☐	☐	☐
63. Sugar-sweetened soft drinks (e.g. Coca-Cola, Fanta, Sprite)	☐	☐	☐	☐	☐	☐
64. Beer	☐	☐	☐	☐	☐	☐
65. Wine and mixed alcoholic drinks	☐	☐	☐	☐	☐	☐

66. Spirits (vodka and other distilled alcoholic beverages)	☐	☐	☐	☐	☐	☐

Thank you for your time and participation.

Food Frequency Questionnaire (FFQ-6)

Terminology and Description of the FFQ-6

The Food Frequency Questionnaire (FFQ-6) was developed to collect information on the frequency of consumption of 62 food groups representing eight major food categories.

1. Sweets and Snacks

- Sugar
- Honey
- Chocolate, chocolate candies and chocolate bars
- Non-chocolate confectionery
- Biscuits, cookies and cakes
- Ice cream and pudding
- Salty snacks

2. Milk and Dairy Products / Eggs

- Milk and plain dairy products
- Sweetened dairy beverages
- Plain cottage cheese and fresh cheeses
- Flavoured cottage cheese
- Cheese
- Eggs and egg-based dishes

3. Cereal Products

- Whole-grain or seeded bread
- Refined bread
- Whole-grain cereals and pasta
- Refined cereals and pasta
- Ready-to-eat breakfast cereals

4. Fats

- Vegetable oils
- Butter
- Margarine
- Cream and sour cream
- Other animal fats
- Mayonnaise and salad dressings

5. Fruits

- Fruits (all types)
- Stone fruits
- Kiwi and citrus fruits
- Other tropical fruits
- Berries
- Bananas
- Apples and pears

- Avocados
- Olives
- Dried fruits
- Sweet fruit preserves and candied fruits

6. Vegetables and Seeds

- Vegetables (all types)
- Cruciferous vegetables
- Yellow-orange vegetables
- Green leafy vegetables
- Tomatoes
- Cucumbers, zucchini, squash and eggplant
- Root and other vegetables
- Fresh legumes
- Dried legumes and legume dishes
- Potatoes
- Nuts and nut spreads
- Seeds

7. Meat and Fish Products

- Sausages
- High-quality cold cuts
- Offal and processed meat products
- Red meat
- Poultry and rabbit meat
- Game meat
- Lean fish
- Fatty fish

8. Beverages

- Fruit juices and fruit nectars
- Vegetable and vegetable-fruit juices
- Energy drinks
- Sugar-sweetened soft drinks
- Beer
- Wine and mixed alcoholic drinks
- Spirits

Questionnaire Description

The FFQ-6 consists of two sections. The first section collects basic participant information, including sex, date of birth and date of examination. The second section assesses the frequency of consumption of 62 food groups during the previous 12 months.

The list of foods and their classification into food groups were developed based on the authors' previous research experience and on the calibrated Food Intake Variety Questionnaire (FIVeQ).

The questionnaire includes two additional verification questions concerning the overall consumption of fruits and vegetables. These questions facilitate interpretation because respondents often overestimate intake when many detailed questions are asked.

Interpretation of Results

The FFQ-6 uses six frequency categories:

- (1) Never or almost never
- (2) Once a month or less
- (3) Several times a month
- (4) Several times a week
- (5) Daily
- (6) Several times a day

The collected data are qualitative and may be analysed using the original six-category scale, collapsed frequency categories, ordinal scores, or converted into semi-quantitative daily frequency values.

Interpreting the FFQ-6 Questionnaire Results

The information obtained is in the format of qualitative data and can be processed and interpreted as follows:

- by applying distribution analysis for the original six food frequency categories,
- by transforming the original food frequency categories into other categories (combining categories),
- by transforming the original food frequency categories into "semi-quantitative" data that logically reflect the increasing intensity of the characteristic (table) by:
  - by ranking them, i.e., assigning conventional integers to the food frequency categories;
  - by transforming them into real numbers and expressing the food frequency as times/day.

Table. Conversion Factors for FFQ-6 Frequency Categories

Frequency category	Assigned rank	Daily frequency (times/day)
Never or almost never	1	0.000
Once a month or less	2	0.025
Several times a month	3	0.100
Several times a week	4	0.571
Daily	5	1.000
Several times a day	6	2.000

Validation of the FFQ-6

The FFQ-6 questionnaire has been validated, and the results have been developed and are currently being prepared for publication (as of March 2, 2014).

The reliability of the FFQ-6 questionnaire was assessed in a group of young girls aged 13 to 21. The reliability of the questionnaire was measured by comparing the results of the initial interview (test) and a repeated interview with the respondent by the same interviewer two weeks later (retest). Spearman's correlation coefficient and kappa statistics were used to assess the reliability of the FFQ-6 questions (test-retest comparison). High Spearman correlation coefficient values (from 0.5 to 0.7) were found for 26 food groups (42% of the total number of products), while very high correlation coefficient values (from 0.7 to 0.9) were obtained for 31 food groups (50% of the total number of products). Kappa statistic values ranged from 0.32 to 0.72. Good test-retest agreement (kappa statistic values from 0.6 to 0.8) was found for 10 food groups (16% of the total number of products), while moderate agreement (kappa from 0.4 to 0.6) was found for 50 food groups (81% of the total number of products). Low test-retest agreement (kappa below 0.4) was obtained for, among others, for root vegetables and other vegetables, ice cream and pudding, ready-made breakfast cereals, natural cottage cheese, dry legumes and legume seeds, meat products and organ meats, and the highest kappa statistic values were obtained for vodka and spirits, wine and mixed drinks, olives, beer, other animal fats, energy drinks, tomatoes, total fruit, green leafy vegetables, chocolate, chocolate candies, and chocolate bars.

How to Cite the FFQ-6

When using the FFQ-6 in scientific publications, please cite the original developers of the questionnaire and the methodological papers describing its development and validation. Until an official English version is published, reference the original Polish FFQ-6 and indicate that the questionnaire was translated for research purposes.

Recommended Citation

- http://www.uwm.edu.pl/edu/lidiawadolowska/

Wądołowska L., Niedźwiedzka E. Food Frequency Questionnaire (FFQ-6). University of Warmia and Mazury, Olsztyn, Poland.

Work is underway to publish the FFQ-6 validation results (Niedźwiedzka E. et al., 2014). Until the validation results are published, the FFQ-6 questionnaire should be cited with the access page, and the validation results provided above (Description of the FFQ-6 validation results) should be included in the methodology of the study.

References

Bland J.M., Altman D.G.: Agreement between methods of measurement with multiple observations per individual. J. Biopharm. Stat., 2007, 17, 571-582.

Cade J., Thompson R., Burley V., Warm D.: Development, validation and utilisation of food-frequency questionnaires – a review. Pub. Health Nutr., 2002, 5, 567-587.

Elmadfa I., Freisling H.: Fat intake, diet variety and health promotion. Forum Nutr., 2005, 57, 1-10.

Horwath C., Kouris-Blazos A., Savige G., Wahlqvist M.L.: Eating your way to a successful old age, with special reference to older women. Asia Pacific J. Clin. Nutr., 1999, 8 (3), 216-225.

Jędrychowski W.: Metoda zbierania wywiadów lekarskich i budowa kwestionariuszy zdrowotnych. PZWL, Warszawa, 1982.

Niedźwiedzka E., Wądołowska L.: Accuracy analysis of the Food Intake Variety Questionnaire (FIVeQ). Reproducibility assessment among older people. Pakistan J. Nutr., 2008, 7, 3, 426-435.

Niedźwiedzka E.: Analiza urozmaicenia spożycia żywności i jego powiązań ze stanem odżywienia i ryzykiem niedożywienia u osób starszych. Rozprawa doktorska, Katedra Żywienia Człowieka, UWM Olsztyn, 2009 (maszynopis).

Niedźwiedzka E., Wądołowska L., Słowińska A., Kwakowicz M.: Kalibracja kwestionariusza urozmaicenia spożycia żywności (FIVeQ) w odniesieniu do metody 7-dniowego bieżącego notowania. Mat. VIII Krajowych Warsztatów Żywieniowych pt. „Metody ankietowe w badaniach żywieniowych", Mrózek k/Olsztynka, 2008, s. 55.

Niedźwiedzka E., Wądołowska L.: Analiza urozmaicenia spożycia żywności i jego powiązań ze stanem odżywienia polskich seniorów. Mat. VI Seminarium Środowiskowego Młodych Pracowników Nauki pt. „Bezpieczeństwo i jakość żywności", Olsztyn, Wyd. UWM, 2009, s. 20-21.

Wądołowska L.: Walidacja metod i mierniki statystyczne w badaniach sposobu żywienia. Rozdz. 3 (w:) Przewodnik metodyczny badań sposobu żywienia. Red. Gronowska-Senger A., Wyd. Komitetu Nauki o Żywieniu Człowieka Polskiej Akademii Nauk, Warszawa, 2013, 17-37.

Wądołowska L.: Zasady obliczania i interpretacji wyników. Rozdz. 4 (w:) Przewodnik metodyczny badań sposobu żywienia. Red. Gronowska-Senger A., Wyd. Komitetu Nauki o Żywieniu Człowieka Polskiej Akademii Nauk, Warszawa, 2013, 38-67. http://www.knozc.pan.pl/index.php/37-uncategorised/89-przewodnik-metodyczny-badan- sposobu-zywienia

Supplementary File S5

DZIENNICZEK BIEŻĄCEGO SPOŻYCIA

Serdecznie proszę o wypełnianie niniejszego dzienniczka, w celu ewentualnej korekty błędów żywieniowych.

Dzienniczek należy wypełniać przez 3 dni w tygodniu – **2 dni powszednie i jeden wolny od pracy (np. sobota/niedziela)**. Proszę o wpisywanie wszystkich zjedzonych potrwa i produktów oraz wszystkich wypitych płynów (także przekąsek, jeśli są).

Prosimy o dokładne opisanie potrawy, np.

- Rodzaj używanego tłuszczu (zwierzęcy, roślinny – nazwa)
- Rodzaj pieczywa
- Sposób przyrządzenia potrawy (gotowanie, pieczenie, smażenie)
- Wszystkie użyte do przyrządzenia potrawy składniki
- Ilość w opakowaniu, gramatura (jeśli to możliwe)

WZÓR:

Godzina	Posiłek	Skład posiłku	Ilość lub miary domowe	Stopień głodu	uwagi
7.30	Śniadanie	Chleb Baltonowski<br>Masło śmietankowe<br>Szynka delikatesowa<br>Pomidor<br>Herbata zielona	2 kromki<br>2 łyżeczki<br>2 plasterki<br>4 plasterki<br>szklanka	1	Herbata bez cukru
9.00	II śniadanie	Baton Mars	1 szt.	5	-

Dzień 1

Godzina	Posiłek	Skład posiłku (produkty)	Ilość lub miary domowe	Pojadanie/uwagi

Dzień 2

Godzina	Posiłek	Skład posiłku (produkty)	Ilość lub miary domowe	Pojadanie/uwagi

Dzień 3

Godzina	Posiłek	Skład posiłku (produkty)	Ilość lub miary domowe	Pojadanie/uwagi

CURRENT FOOD INTAKE DIARY

Please complete this diary for 3 days (2 weekdays and 1 weekend day). Record all foods, beverages and snacks consumed. Describe meals in detail, including the type of fat, bread, cooking method, ingredients, and portion size whenever possible.

Day of the week: _____________ Date: _____________

Name and surname: ________________________________

Example

Time	Meal	Food items / Products	Amount or household measures	Hunger level	Comments
7:30	Breakfast	Baltonowski bread<br>Butter<br>Ham<br>Tomato<br>Green tea	2 slices<br>2 tsp<br>2 slices<br>4 slices<br>1 cup	1	Tea without sugar
9:00	Morning snack	Mars bar	1 bar	5	–

Day 1

Time	Meal	Food items (products)	Amount or household measures	Snacking / Comments

Day 2

Time	Meal	Food items (products)	Amount or household measures	Snacking / Comments

Day 3

Time	Meal	Food items (products)	Amount or household measures	Snacking / Comments
